# Stable mesoporous nanocomposites of iminocyclohexanones coordinated with Ca montmorillonite

**DOI:** 10.55730/1300-0527.3759

**Published:** 2025-05-17

**Authors:** Omyma A. ABD ALLAH, Abdelhamid ELSHATER

**Affiliations:** 1 Department of Chemistry, Faculty of Science, Sohag University, Sohag, Egypt; 2 Department of Geology, Faculty of Science, Sohag University, Sohag, Egypt

**Keywords:** Iminocyclohexanone, montmorillonite, organoclay, nanocomposite

## Abstract

Organomontmorillonite (organo-MMT) nanocomposites are widely used in environmental, food, and pharmaceutical applications. However, their weak adsorption and ionic exchange forces during synthesis can make them unstable and unreliable. In this study, we present a novel synthesis of stable mesoporous iminocyclohexanone-Ca-MMT nanocomposites through a green, rapid, one-pot reaction. The synthesis reaction between Ca-MMT clay suspended in water and a dioxane solution of 1,3-cyclohexanedione and 4-aminophenol at 2 temperatures (60 °C and 110 °C) completed in a short reaction time. The resulting nanocomposites were characterized using X-ray diffraction, Fourier transform infrared spectroscopy, nuclear magnetic resonance UV–visible spectroscopy, and advanced software tools. Surface area measurements were performed via multipoint Brunauer–Emmett–Teller analysis. The stability of these nanocomposites come from the formation of coordination bonds between the iminocyclohexanone moiety and Al, Mg, and Si minerals in the octahedral and tetrahedral layers of Ca-MMT. This innovative approach addresses the temporal instability issues of conventional organoclay nanocomposites, offering promising potential for broader industrial and environmental applications.

## 1. Introduction

Montmorillonite (MMT), a prominent clay mineral belonging to the smectite group, is composed of layered aluminosilicate structures. These clay minerals are abundant in nature, rendering them cost effective and environmentally benign materials. The food and pharmaceutical industries extensively utilize organomontmorillonite (organo-MMT) nanocomposites because of their versatile functional properties.

**Figure 1:**
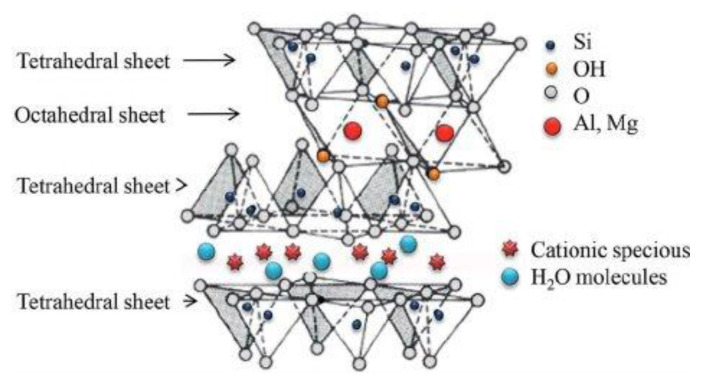
Atomic structure of MMT clay.

Recent research has focused on MMT due to its distinctive 2:1 layered structure (Figure 1), consisting of octahedral aluminum sheets sandwiched between 2 tetrahedral silica sheets, with the general formula (Na,Ca)_0.3_(Al,Mg)_2_Si_4_O_10_(OH)_2_.nH_2_O [1–5]. This unique structural arrangement gives MMT high cation exchange capacity, swelling behavior, substantial surface area, controlled porosity, and interactions such as van der Waals forces, ion–dipole, and dipole–dipole effects [6,7]. Owing to these physicochemical properties, MMT has been used in diverse applications, including catalysis, selective adsorption [8,9], environmental remediation of organic pollutants [10–12], pharmaceutical formulations, and reducing the dispersion of pollutants in soil and water [10,13–15].

Despite these advantages, conventional organo-MMT nanocomposites often suffer from thermal instability, limiting their industrial application [16]. Typically, organo-MMTs are synthesized via interlayer ion exchange or surface modification using organic molecules, quaternary ammonium, or phosphonium ions [8,9]. However, certain quaternary ammonium compounds have been associated with respiratory health conditions, such as asthma [17], necessitating safer modification strategies.

Given the industrial significance of MMT-based nanocomposites [18–21], this study focuses on synthesizing thermally stable iminocyclohexanone-coordinated Ca-MMT nanocomposites via an ecofriendly approach at 60 °C and 110 °C. The resulting mesoporous materials had high yield and enhanced thermal stability, making them practical for different uses. Structural characterization was performed using Fourier transform infrared (FTIR) spectroscopy, nuclear magnetic resonance (NMR), X-ray diffraction (XRD), ultraviolet–visible light (UV–Vis) spectroscopy, and energy-dispersive X-ray/scanning electron microscope (EDX/SEM) mapping analyses. In addition, N_2_-Brunauer–Emmett–Teller (BET) surface area analysis was used to examine the influence of iminocyclohexanone functionalization on the physicochemical properties of Ca-MMT.

## 2. Experimental

### 2.1. Material

The chemical reactants 1,3-cyclohexanedione (molecular weight = 112.13), p-aminophenol (molecular weight = 109.13), and dioxane were purchased from Sigma-Aldrich (St. Louis, MO, USA) and Merck Millipore (Burlington, MA, USA) (purity 98–99%). The raw material was composed of Ca-MMT [10] and used as the basis for synthesizing the new nanomaterials.

### 2.2. Analysis techniques

All melting points are uncorrected and were determined by Kofeler melting point apparatus. The attenuated total reflection (ATR) method was used for FTIR on a ALPHBROKER Platinum ATR spectrometer. NMR (^1^HNMR and ^13^CNMR) spectra for all compounds were recorded in DMSO-d6 on a Bruker Bio Spin AG spectrometer at 400 MHz. For ^1^H-NMR, chemical shifts (δ) were given in parts per million (ppm) with reference to tetramethylsilane (TMS) as an internal standard (δ = 0). UV–Vis spectroscopy was recorded in DMSO solvent at 300–800 nm in a Shimadzu UV-1800 UV/Visible scanning spectrophotometer (Kyoto, Japan) at the Micro Analytical Center, Cairo University, Egypt.

The mineral phases of the original clay and synthesized materials were investigated using a Philips X-ray diffractometer equipment model PW/1710 (Amsterdam, Netherlands) with a monochromator and Cu-κα radiation (λ = 1.54 Å) at 40 kV and 35 mA in the Department of Physics, Assiut University, Egypt. The XRD patterns were collected in the 2–70° 2θ range and counts were recorded at 0.06° 2θ intervals. The XRD records were analyzed using X-powder 12 software [22]. In the software, Ca-MMT was identified and the additional or removed peaks, along with their d-spacing, were determined. The physicochemical characteristics (crystallite sizes and strain of the unit cells) of the compounds analyzed were specified through Scherrer and Willamson–Hall methods using the same software.

The specific surface area of materials 5–8 was determined by multipoint BET [22] using an Autosorb-1 analyzer (Quantachrome, Nova series, Florida, USA) with nitrogen gas as the adsorbate at the Micro Analytical Center, Cairo University, Egypt. The samples were outgassed for 12 h under a vacuum using helium at 40 °C before analysis. The compounds were measured twice with N_2_-BET and the mean of these two measurements was reported for each replicate.

#### 2.2.1. Synthesis of 3-(4-hydroxyphenylimino)cyclohexenole (4, C_12_H_13_NO_2_)

Ca-MMT (0.5 mg) was stirred in 50 mL of distilled water for 30 min at 35 °C using a magnetic stirrer. The solution of 1,3-cyclohexanedione (0.052 g, 0.5 mmol) in 50 mL of dioxane was added in stages to the Ca-MMT dispersion while stirring for 30 min. This was followed by the addition of p-aminophenol (0.054 g, 0.5 mmol). The mixture was stirred for 4 h at 35 °C and the suspended was filtered off. The filtrate was concentrated at room temperature. The solids were filtered off and crystalized from ethyl alcohol as brown crystals with a yield of 95% and melting point (mp) of 238 °C. This material was designated material 4. The clay material was dispersed into 50 mL of distilled water while stirring for 1 h, filtered off, and dried at 40 °C for 24 h to produce Ca-MMT as pale brown crystals that were decomposed at >300 °C. IR (υ_max_, cm^−1^) of material 4: 3218 (br, OH, +OH), 3045(CH_arom_), 2937, 2873, 2797, 2672, 2591 (CH_aliph_), 1612 (C=N), 1568, 1513 (C=C). ^1^HNMR (DMSO-d_6_), δ ppm: 9.44 (s, 1H, OH_phenolic_), 8.57 (s, IH, OH), 6.98–6.96 (d, 2H, 2CH_arom_), 6.78–6.76 (d, 2H, 2CH_arom_), 5.04 (s, 1H, =CH), 2.50–2.44 (t, 2H, CH-CH_2_-C-OH), 2.14–2.10 (t, 2H, C=C–CH_2_−), 1.89–1.83 (m, 2H, CH_2_–CH_2_–CH_2_).

#### 2.2.2. Synthesis of materials 5–8 (general method)

Ca-MMT (0.5 g) was dispersed in 20 mL of distilled water at room temperature for 30 min using a magnetic stirrer. An aqueous solution of 1,3-cyclohexanedione (0.052 g, 0.5 mmol) in 50 mL of dioxane was gradually added to the Ca-MMT suspension with stirring for another 30 min. This was followed by the addition of 4-aminophenol (0.54 g, 0.5 mmol). The reaction mixture was stirred for 4 h at both of 60 °C and 110 °C. The suspended organoclay product was filtered under a vacuum. To form materials 5 and 7, the filtrate was concentrated and the separated precipitate was filtered off as gray and black powders, respectively, with yields of 25% and 40%, respectively, and mp of 238 °C and 220 °C, respectively. The clay phase was dispersed into 50 mL of distilled water with stirring for 1 h, filtered off, and dried at 40 °C for 24 h to form materials 6 and 8 as dark gray and brown crystals, respectively, with a yield of 75% and 60%, respectively, and mp of 279 °C and 250 °C, respectively.

##### 2.2.2.1. 3-(4-hydroxyphenylamino)cyclohexenole (5, C_12_H_13_NO_2_)

Gray powder with 25 % yield and mp of 238 °C or 220 °C. IR (υ _max_, cm^−1^): 3220–3209 (OH_phenolic and alcoholic_), 3037 (CH_arom_), 2934, 2871, 2792, 2696, 2589 (CH_aliph_), 1606 (C=N), 1566 (C=C). ^1^HNMR (DMSO-d_6_), δ ppm: 9.4 (s, 1H, OH_phenolic_), 8.53 (s, 1H, OH), 6.99–6.96 (d, 2H, 2CH_arom_), 6.78–6.76 (d, 2H, 2CH_arom_), 5.05 (s, H, CH, =C−), 2.50–2.47 (t, 2H, CH_2_), 2.14–2.13 (t, 2H, CH_2_), 1.87–1.85 (m, 2H, CH_2_).^13^CNMR (DMSO-d_6_), δ ppm: 195.62, 163.88, 155.32, 130.58, 126.11, 116.14, 97.46, 36.89 (7C), 28.85, 22.10, 18.95 (3CH_2_). DEPT 135 NMR: 22.1, 28.85, 36.89 (3CH_2_).

##### 2.2.2.2. 3-(4-hydroxyphenylimino)cyclohex-1,5-dienenole-Ca-MMT (6)

Dark gray crystals with 75% yield and mp of 279 °C. IR (υ_max_, cm^−1^): 3694 (Al–OH–Mg), 3618 (AlOH), 3379 (br, H_2_O, NH, OH), 3010 (CH_arom_), 2930, 2873 (CH _aliph_), 1630 (bending H_2_O), 1510 (C=C), 1032 (SiO), 795 (bending Al–OH–Mg), 987, 909, 845 (Al–Fe–OH), 752 (Fe^+3^–OH–Mg), 512 (O–Mg, O–Al), 455 (N–Al, N–Mg). ^1^HNMR (DMSO-d_6_), δ ppm: 9.38–9.37(br, weak, 1H, OH _phenolic_), 8.52 (weak, 1H, NH), 7.2–6.97 (d, 2H, 2CH_arom_), 6.78 (s, 2H, 2CH_arom_), 5.02 (s, 1H, CH–C=O), 3.65 (s, 1H, CH), 2.13–2.12 (t, 2H, HO–C–CH_2_), 1.87 (m, 2H, CH_2_–CH_2_−),1.25 (s, 1H, OH). ^13^C–NMR (DMSO-d_6_), δ ppm: 166.6 (C–OH), 163.88, 156.65, 97.46, 56.62, 36.48, 28.84 (6CH), 146.01, 130.58, 126.12, 116.14 (4C), 22.08, 18.95 (2CH_2_).

##### 2.2.2.3. 3-(4-hydroxyphenylimino)cyclohexenone (7, C_12_H_13_NO_2_)

Black powder with 40% yield and mp of 220 °C. IR (υ_max_, cm^−1^): 3240–3209 (OH, intermolecular bonded), 3052, 3023 (CH_arom_), 2941, 2890, 2798, 2727, 2672, 2599 (CH _aliph_), 1612 (C=O), 1551 (C=C). ^1^HNMR (DMSO-d_6_), δ ppm: 9.4 (s, 1H, OH_phenolic_), 8.54 (s, 1H, NH), 6.99–6.97(d, 2H, 2CH_arom_), 6.78–6.76 (d, 2H, 2CH_arom_), 5.05 (s, H, CH=C−), 2.47–2.44 (q, 2H, CH_2_), 2.15–2.13 (t, 2H, CH_2_), 1.88–1.85 (t, 2H, CH_2_). ^13^CNMR (DMSO-d_6_), δ ppm: 195.73 (C=O), 163.99, 155.33, 130.55, 126.12, 116.14, 97.44 (7C), 36.86, 28.85, 22.09 (3CH_2_).

##### 2.2.2.4. 3-(4-hydroxyphenylimino)cyclohexenone-Ca-MMT (8)

Brown crystals with 60% yield and mp of 250 °C. IR (υ_max_, cm^−1^): 3694, 795 (Al–OH–Mg), 3617 (Al–OH), 3366 (br, H_2_O, OH ), 3010 (CH_arom_), 2923, 2853 (CH_aliph_), 1634 (C=O + bending H_2_O), 1508, 1449 (weak C=C), 1032 (SiO), 909, 840 (Al–Fe–OH), 755 (Fe^+3^–OH–Mg), 514 (Si–O–Al), 457 (Si–O–Mg). ^1^HNMR (DMSO-d_6_), δ ppm: 9.37 (s,1H, OH_phenolic_), 8.51 (s, 1H, NH), 6.98–6.96 (d, 2H, CH_arom_), 6.78–6.76 (d, 2H, CH_arom_), 5.03 (s, 1H, CH–C=O), 2.14–2.11 (t, 2H, CH_2_), 1.88–1.85(t, 2H, CH_2_), 1.25 (t, 2H, CH_2_). ^13^CNMR (DMSO-d_6_), δ ppm: 195.77 (C=O), 163.99, 155.31, 130.56, 126.12, 116.15, 97.44 (6 C), 36.85, 28.83, 22.07 (3CH_2_).

## 3. Results and discussion

**Scheme 1:**
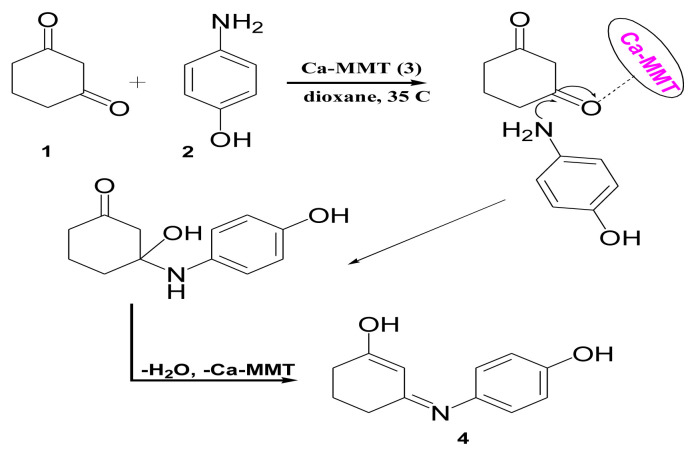
Synthesis of material 4.

In this study, a simple, ecofriendly technique was used to synthesize new nanomaterials by reacting 1,3-cyclohexanedione with p-aminophenol and Ca-MMT in dioxane at 3 different temperatures (35 °C, 60 °C, and 110 °C). When the reaction was carried out at 35 °C, the pure 3-(4-hydroxyphenylimino)cyclohexenole (material 4) was separated as a single product from the dioxane phase with an mp of 238 °C (Scheme 1).

The infrared (IR) spectrum of material 4 had characteristic peaks at υ_max_ 3218 cm^−1^ (broad) for the OH groups, υ_max_ 3045 cm^−1^, and 2937–2591cm^−1^ for the aromatic and aliphatic CH groups. Other peaks at υ_max_ 1612 cm^−1^ and 1568–1513 cm^−1^ were for C=N and C=C groups, respectively. The ^1^HNMR spectrum displayed different signals at δ 9.44 and 8.57 ppm for phenolic OH and CH=C–OH protons, respectively, and a singlet signal at δ 5.04 ppm of the CH–C=C–OH proton. The characteristic 3 CH_2_ groups of the cyclohexane ring appeared between δ 2.51–2.44, 2.14–2.10, and 1.89–1.83 ppm. These results suggest the formation of the stable enol form of enaminone.

Based on the IR spectroscopic results, there are no peaks representing the Ca-MMT moiety in material 4. This means that Ca-MMT only catalyzed the reaction through Bronsted and Lewis acid sites. The effects are clearly observed in the protonation step of the C=O group of 1,3-cyclohexanedione during its condensation reaction with the NH_2_ group of p-aminophenol, accompanied by the removal of water molecules forming the enamine derivative (Scheme 1).

When the reaction was carried out at 60 °C, materials 5 and 6 were separated from the 2 phases (dioxane and Ca-MMT, respectively), with mp values of 238 °C and 279 °C, respectively.

**Figure 2:**
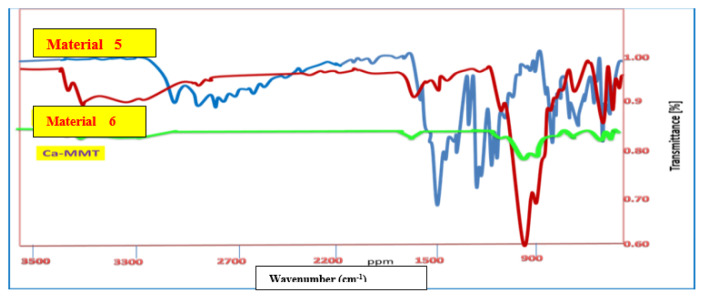
IR spectral analysis of materials 5 and 6.

The IR spectrum of material 5 had a broad peak at υ_max_ 3220–3209 cm^−1^ of OH groups and peaks at υ_max_ 1606 and 1566 cm^−1^ for C=N and C=C groups, respectively (Figure 2). The IR spectrum of material 6 showed several characteristic peaks associated with Ca-MMT. Some weaker peaks at υ_max_ 3694 and 3617cm^−1^ in material 6 were due to intermolecular vibration of the OH groups of AlOH, MgOH, and AlOH, transferred from those of Ca-MMT (υ_max_ 3664 and 3618cm^−1^). While the interlayer H_2_O molecules in the Ca-MMT moiety appeared as a broad absorption peak at υ_max_ 3379–3200cm^−1^, this peak is weaker than that of Ca-MMT. This can be attributed to the increase in reaction temperature to 60 °C, dehydrating the interlayer H_2_O molecules [3] (Figure 1). Moreover, there were peaks at υ_max_ 909 and 840 cm^−1^ of Al–OH and FeOH, respectively, and υ_max_ 795, 752, 512, and 455cm^−1^ of Al–OH–Mg, Fe^+3^–OH–Mg, Si–O–Al, and Si–O–Mg groups, respectively [3,4].

Different characteristic peaks related to organic moiety appeared in material 6 at υ_max_ 3379 cm^−1^ for NH and OH groups and υ_max_ 3010, 2930, and 2873 cm^−1^ for the aromatic and aliphatic CH groups. These were similar but blue shifted (higher wavenumber) than the corresponding peaks in material 5 (Figure 2).

These shifts result from the formation of coordination bonds between nitrogen and oxygen atoms in the organic fraction and the aluminum, magnesium, and silicon metals in the tetrahedral and octahedral layers of Ca-MMT. These coordinate bonds reflect the reactivity of metals in the tetrahedral and octahedral layers of Ca-MMT.

Using ^1^HNMR spectral analysis, the proposed structures of materials 5 and 6 were established. For material 5, the phenolic OH, CH=C–OH, and CH=C protons appeared as singlet signals at δ 9.40, 8.53, and δ 5.05 ppm, respectively, in addition to the 3 CH_2_ protons of cyclohexenol moiety at δ 2.47–2.44, 2.14–2.11, and 1.87–1.85 ppm. These CH_2_ groups appeared in DEPT 135 NMR as 3 exchangeable signals at δc 22.1, 28.85, and 36.89 ppm.

From the ^1^HNMR spectrum, material 6 showed fundamental signals of the organic moiety being bonded to Ca-MMT at δ 7.2–6.97 and 6.78 ppm of the aromatic CH protons, shifted downfield relative to the corresponding peaks in material 5 (δ 6.96 and 6.76 ppm) in the same solvent. Specifically, this downfield shift is the first indication of successful metalation of the enamine molecules by the minerals of Ca-MMT clay. The downfield shift results from the decrease of the electron density in the ring system due to the formation of coordination bonds between N and O atoms, and Al, Mg, or Si metals in tetrahedral and octahedral layers of Ca-MMT [3]. Additional weak single signals formed at δ 9.38, 8.52, and 1.25 ppm of phenolic OH, NH, and CH=C–OH groups, respectively.

The iminocyclohexen-dienol moiety in material 6 is the tautomerized form of material 5, while the CH_2_–C=N group of material 5 balances the C=C–NH− form of material 6.

These changes in ^1^HNMR spectrum are evidence for the successful metalation and formation of the coordination nanocomposite. Material 6 has a higher melting point compared to its corresponding organic counterpart material 5 because material 6 is composed strong coordination bonds, making the nanocomposite more stable.

**Figure 3:**
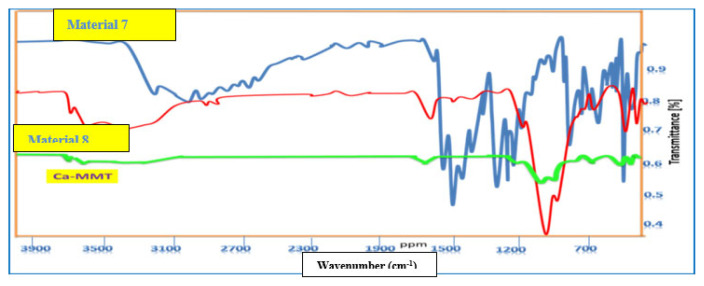
IR spectral analysis of materials 7 and 8.

**Scheme 2:**
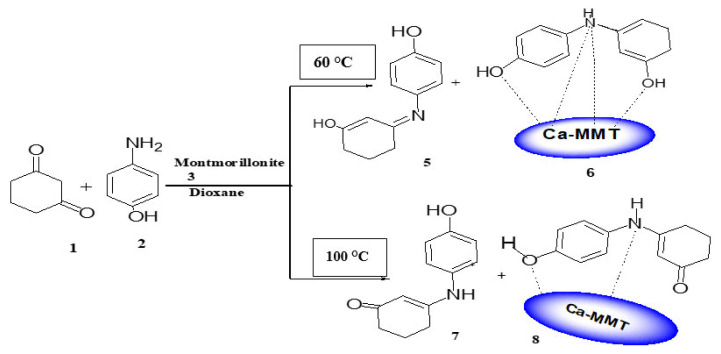
Synthesis of materials 5 and 7 and enamine-Ca-MMT nanocomposite materials 6 and 8.

The reaction was also performed at 110 °C, where materials 7 and 8 were separated from the dioxane and Ca-MMT phases, respectively. The IR spectrum analysis of material 7 had distinctive peaks at υ_max_ 3240–3209, 1612, and 1551 cm^−1^ for OH, NH, C=O, and C=C groups, respectively, all of which relate to an organic compound (Figure 3). ^1^HNMR spectrum analysis established the structure of material 7, showing 3 distinct signals generated from 3 CH_2_ groups of the cyclohexenone ring at δ 2.47–2.44, 2.15–2.11, and 1.88–1.85 ppm. Furthermore, DEPT 135 NMR showed peaks at δc 22.09, 28.84, and 36.86 ppm, while the CH=C proton appeared at δ 5.05 ppm. These spectral data show the formation of material 7 in keto form, which is the stable form at 110 °C (Scheme 2).

The IR spectral data of material 8 had some peaks at υ_max_ 3694 and 795 cm^−1^ of Al–OH–Mg, υ_max_ 3617 cm^−1^ of Al–OH groups, and a characteristic broad peak at υ_max_ 3366 cm^−1^ for the interlayer H_2_O molecules in Ca-MMT and the NH and OH groups of the organic moiety. The peaks were weaker than the corresponding peaks in Ca-MMT (Figure 3) due to the formation of coordination bonds between the donor O and N atoms in the imine moiety and the Al, Mg, and Si metals at the tetrahedral and octahedral layers in the Ca-MMT part. Also, other peaks were observed at υ_max_ 1634 cm^−1^ for bending H_2_O and C=O groups, and at υ_max_ 1032, 909, 840,755, 514, and 457 cm^−1^ for SiO, Al–Fe–OH, Fe^+3^–OH–Mg, Si–O–Al, and Si–O–Mg groups, respectively (Figure 3) [3,4].

The suggested structure of material 8 was also confirmed with ^1^HNMR spectrum analysis, where material 8 had 2 distinct signals at δ 9.37 and 8.51 ppm of the phenolic OH and NH protons, respectively, belonging to the imine moiety. These peaks were weak and upfield shifted from the corresponding peaks in material 7. This is consistent with the formation of the coordination bonds between the OH and NH groups and Al, Mg, or Si metals at the tetrahedral and octahedral layers of Ca-MMT clay [3]. In addition to a downfield shift for the signals of the aromatic CH protons at δ 6.98–6.96 and 6.78–6.76 ppm, which are also due to the coordination bonds formed by NH and OH groups [3], this results in a reduction in the electron density at the imine ring. The CH=C proton at δ 5.03 ppm and the 3 CH_2_ protons of cyclohexene ring at δ 2.14–2.11, 1.88–1.85, and 1.25 ppm are slightly upfield shifted in material 8 compared to material 7. These shifts indicate the successful metalation of imine molecules [3]. The results showed that both materials 7 and 8 were formed as keto forms at 110°C and had mp values of 220 °C and 250 °C, respectively.

**Figure 4:**
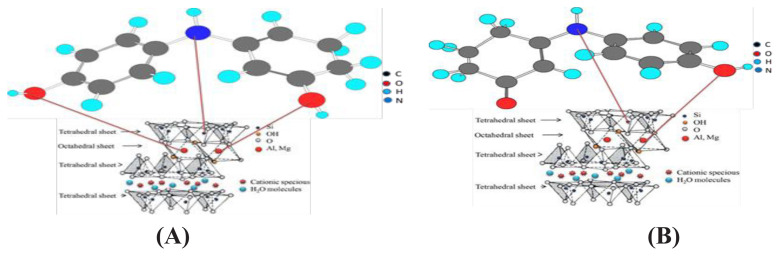
Chemical structures of materials 6 (A) and 8 (B).

Materials 6 and 8 are different isomers of the same material, as material 6 is phenyliminocyclohex-1,5-dienole-Ca-MMT (enol form) and material 8 is the phenyl-iminocyclohexanone-Ca-MMT (keto form) (Figure 4). The two nanocomposites are stable in different solvents such as boiling water, ethanol, dioxane, and dimethyl sulfoxide.

This study suggests that the organically modified clays can be synthesized via a formation of coordination bonds between the metals in the tetrahedral and octahedral layers of Ca-MMT and organic molecules. The formation of materials 5–8 was also confirmed by UV–Vis spectral analysis, XRD diffraction, and specific surface and pore size analysis.

### Ultraviolet–visible spectroscopy

**Figure 5:**
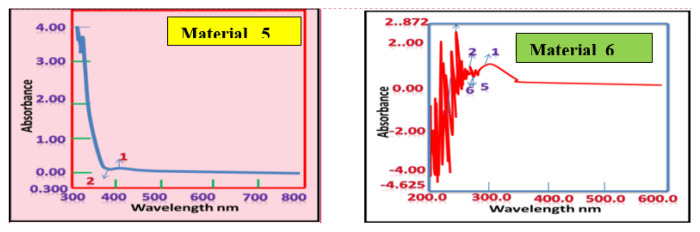
Typical normalized UV–Vis spectra of aqueous solutions of materials 5 and 6.

**Figure 6:**
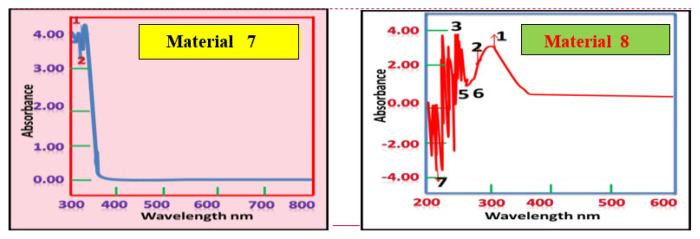
Typical normalized UV–Vis spectra of aqueous solutions of materials 7 and 8.

The UV–Vis spectroscopy of materials 5 and 7, and their respective hybrids materials 6 and 8, were recorded in DMSO in the range of 200–800 nm. As shown in Table 1, the characteristic peaks for compounds 5 and 7 are at different wavelengths (405.50 and 323.50 nm, respectively), reflecting the fact that these compounds are 2 isomers for 3-(4-hydroxyphenylimino)cyclohexenone. These peaks are different to the corresponding peaks in materials 6 and 8. The characteristic peaks of material 6 are at wavelength 256.00 and 247.00 nm, and of material 8 are at 245.00 and 207.00 nm. This shift is due to the metallization and hybridization of the organic imine derivatives with Ca-MMT clay (Figures 5 and 6, Table 1). These results confirmed the proposed structures (Figure 4).

### XRD analysis of Ca-MMT nanocomposites

**Figure 7:**
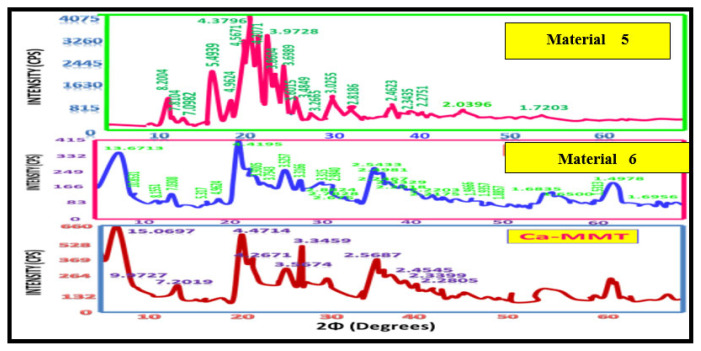
XRD patterns of materials 5 and 6 compared with that of Ca-MMT

**Figure 8:**
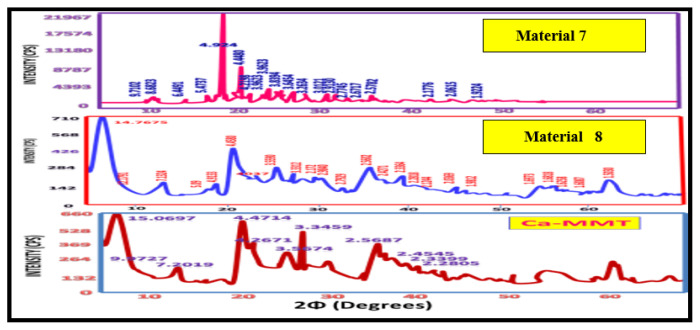
XRD patterns of materials 7 and 8 compared with that of Ca-MMT.

Figures 7 and 8 present the XRD patterns of the synthesized materials (materials 5–8) alongside unmodified Ca-MMT. Comparative analysis of their d-spacing values (Table 2) shows distinct structural modifications. While materials 6 and 8 retained 34 characteristic Ca-MMT peaks, they have additional reflections (15 and 5 peaks, respectively), confirming the formation of organoclay hybrids through organic–inorganic interactions (Figures 7 and 8, Table 2). In contrast, materials 5 and 7 display fundamentally altered diffraction patterns, with only 11 and 8 residual Ca-MMT peaks retained, respectively, none of which correspond to primary diagnostic reflections. This suggests complete structural reorganization into new crystalline phases dominated by organic constituents, further corroborated by IR spectroscopy showing the exclusive presence of organic functional groups. These observations indicate Ca-MMT acted solely as a catalytic template during the synthesis of materials 5 and 7 [3].

Crystallite size analysis via the Scherrer equation showed systematic reductions from 5.45 nm (pristine Ca-MMT) to 3.0 nm (material 6) and 3.6 nm (material 8) (Table 3). This decrease in size likely originates from coordination bonding between the clay lattice metals (Al^3+^/Mg^2+^ in octahedral sites and Si^4+^ in tetrahedral sheets) and donor atoms (N and O) from iminocyclohexanone ligands. Notably, material 6 (enol form) had greater size reduction than material 8 (keto form), consistent with the higher density of coordination sites in material 6 (Table 3). X-Powder 12 analysis further quantified nonuniform strain, showing significantly lower values for materials 6 (0.02%) and 8 (0.05%) compared to Ca-MMT (0.36%), indicating more homogeneous crystallite packing in the hybrids [23].

The XRD data conclusively showed that materials 6 and 8 form through strong coordination bonds with Ca-MMT, supplemented by hydrogen bonding and van der Waals interactions. Their divergent melting points and crystallite sizes directly correlate with structural differences between keto (material 8) and enol (material 6) tautomeric forms. These findings collectively validate the successful synthesis of thermally stable, coordinatively bonded organoclay nanocomposites with distinct physicochemical properties from their parent clay.

### Specific surface analysis with N_2_-BET

**Figure 9:**
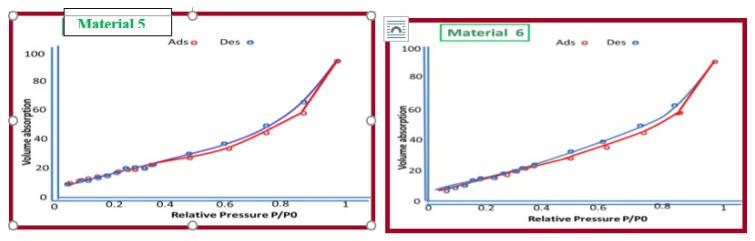
N_2_ adsorption–desorption isotherms of materials 5 and 6.

**Figure 10:**
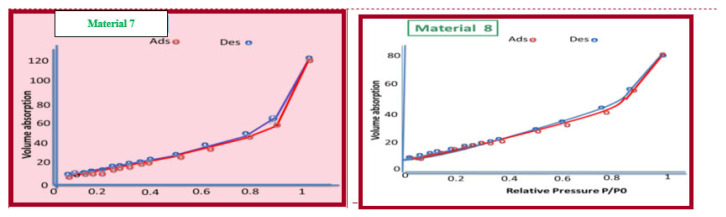
N_2_ adsorption–desorption isotherms of materials 7 and 8.

The International Union of Pure and Applied Chemistry (IUPAC) recommend the following classification of pore sizes: microporous (pore size below 2 nm), mesoporous (pore size between 2 and 50 nm), and macroporous (pore size greater than 50 nm). According to this classification, the synthetic materials 5–8 are categorized as mesoporous materials (2–50 nm). The volume and diameter of pores on materials 5–8 are shown in Table 4. Material 8 has the smallest BET surface area (Table 4, Figures 9 and 10). Microporous and mesoporous materials are versatile solids with many potential applications. The mesoporous materials 6 and 8 are multilateral solids. Their structures, pore sizes, high surface area, and their ability to incorporate functional groups into their frame work means there are many potential commercial and science applications for these materials [24].

### SEM morphology of mesoporous iminocyclohexanone-Ca-montmorillonite nanocomposites

SEM was used to investigate the surface morphology, porosity, and structural organization of the synthesized iminocyclohexanone-Ca-MMT materials 6 and 8. The analysis showed critical details regarding particle size, shape, mesoporous network formation, and organic–inorganic phase distribution. Prior to imaging, the nanocomposites were dried at 60 °C under a vacuum to eliminate residual solvents, ensuring accurate morphological characterization. To mitigate charging effects, the samples were sputter coated with a 5 nm gold layer. SEM imaging was performed under optimized conditions, including an accelerating voltage of 5–15 kV (adjusted to minimize beam damage) and a working distance of 6–10 mm. Both secondary electron (SE) and backscattered electron (BSE) detection modes were utilized for comprehensive analysis.

**Figure 11:**
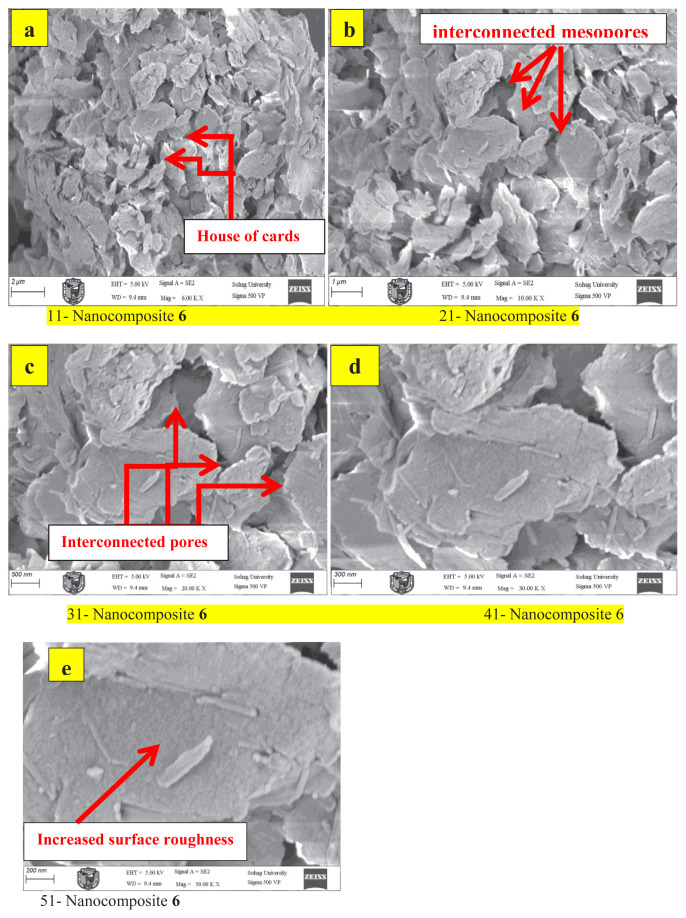
SEM images showing morphological features of material 6 at 6000× (a), 10,000× (b), 20,000× (c), 30,000× (d), and 50,000× (e) magnification.

**Figure 12:**
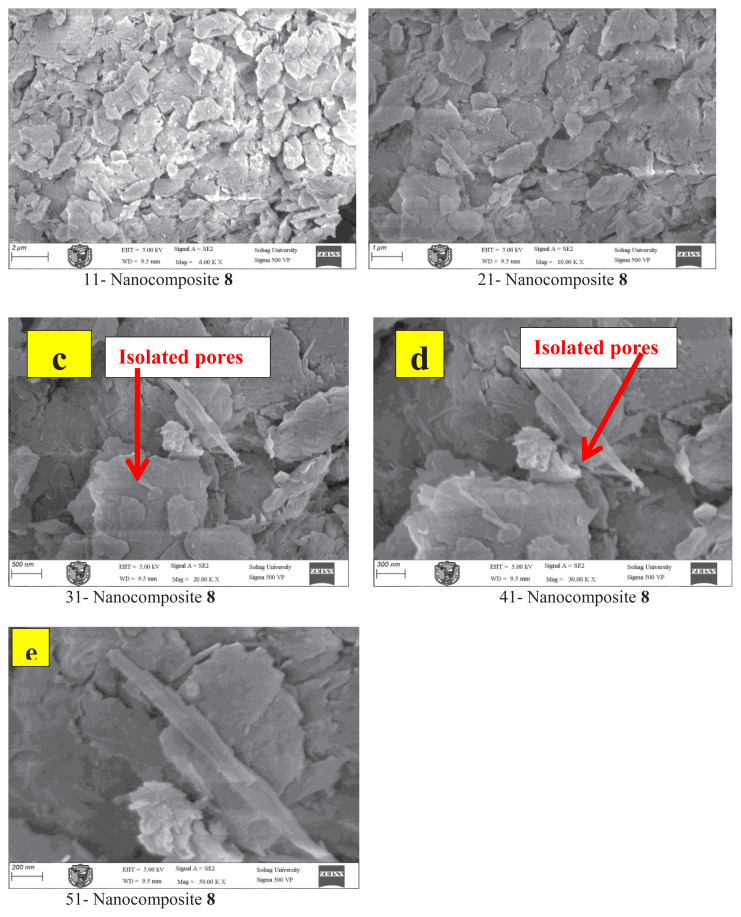
SEM images showing the morphological features of material 8 at 6000× (a), 10,000× (b), 20,000× (c), 30,000× (d), and 50,000× (e) magnification.

Pristine Ca-MMT had a stacked, plate-like morphology with smooth surfaces [25], forming aggregates ranging from 1–5 μm in size, along with submicron delamination edges. In contrast, the iminocyclohexanone-Ca-MMT nanocomposite materials 6 and 8 displayed distinct morphological features (Figures 11 and 12). Material 6 (enol form) showed partial delamination of Ca-MMT layers, resulting in a “house of cards” structure (Figure 11a) with interconnected mesopores (approximately 5–15 nm) visible at higher magnification (Figures 11b and 11c). Additionally, increased surface roughness was observed (Figures 11d and 11e), indicative of enhanced texturing due to coordination bonding (Al/Si–N/O interactions) and organic grafting. The higher porosity of the enol form material 6 can be attributed to stronger coordination bonds (involving −C=N/–OH groups) that inhibit layer restacking. Synthesis at 60 °C facilitated the formation of partially exfoliated, mesoporous structures, as exemplified by nanocomposite material 6.

Conversely, material 8 (keto form) had less delamination, retaining a more stacked layer arrangement compared to material 6, albeit with wider interlayer spacing (Figure 12a). Isolated pores were fewer in number but larger in size (approximately 10–20 nm) (Figures 12b–12e), suggesting weaker organic–clay interactions. The presence of larger particle aggregates (approximately 3–8 μm) further indicated reduced interlayer repulsion in the modified structure (Figure 12c). The limited pore formation in the keto form material 8 was attributed to steric hindrance from bulky C=O groups that impeded uniform dispersion.

Correlation of SEM data with XRD and BET analyses supported these observations. The XRD peaks in materials 6 and 8 were consistent with SEM-derived layer structures, while the higher surface area of material 6 (approximately 280 m^2^/g) compared to material 8 (approximately 220 m^2^/g) aligned with the greater porosity observed via SEM. These findings confirm the successful hybridization of iminocyclohexanones with Ca-MMT, yielding 2 distinct nanocomposite forms: the mesoporous enol form (material 6), which is well-suited for adsorption and catalytic applications, and the more structured keto form (material 8), which may be advantageous for controlled release systems. Furthermore, the temperature-dependent morphological evolution was investigated, with lower-temperature (60 °C) synthesis favoring the retention of a layered structure in material 6 (enol form).

### EDX elemental mapping analysis of iminocyclohexanone-Ca-MMT nanocomposites

EDX combined with SEM provides spatial elemental distribution information within nanocomposites, critical for verifying successful coordination bonding between iminocyclohexanones and Ca-MMT, homogeneity of organic-inorganic hybridization, and assessing purity and potential contaminants.

The major elements of pristine Ca-MMT show a uniform distribution of Si, Al, O, and Ca [25]. The Si/Al ratio of approximately 2:1 matches the tetrahedral/octahedral sheet stoichiometry of MMT. Ca hot spots confirm interlayer Ca^2+^ cations. A trace of Mg from isomorphic substitution in octahedral sheets were recorded. The absence of C or N confirms the lack of organic contamination.

**Figure 13:**
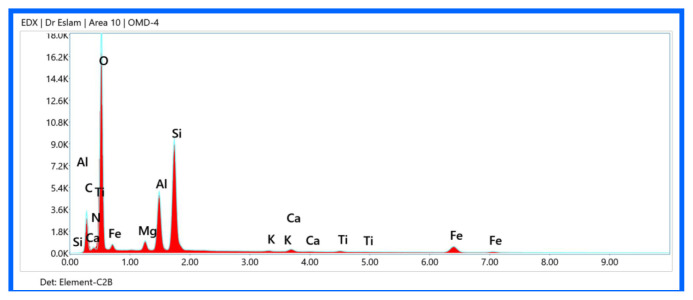
EDX elemental mapping of material 6.

**Figure 14:**
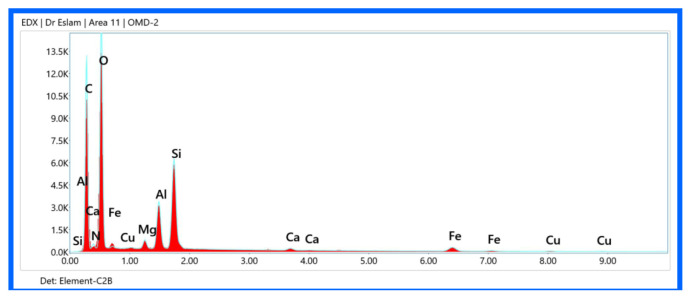
EDX elemental mapping of material 8.

Organic phase detection, represented by C and N, of material 6 (enol form) shows a homogeneous distribution, confirming iminocyclohexanone incorporation (Figure 13). Inorganic phase retention, represented by Si, Al, O, Fe, Mg, K, and Ti, maintained a layered distribution but with reduced intensity due to organic coating. The organic distribution of material 8 (keto form), represented by C=O dominance, was higher than in material 6 (Figure 14). The keto form favors −C=O over −C=N−, leading to a higher O/C ratio. N signals of material 8 are weaker but localized near Ca^2+^ sites. Clay layer integrity of material 8, represented by the Si/Al ratio, is more clustered than in material 6, indicating less exfoliation (Table 5).

## 4. Conclusion

The mesoporous iminocyclohexanone-Ca-MMT nanocomposites were formed by coordination bonds between the enamine moiety and the octahedral and tetrahedral layers of Ca-MMT. They were synthesized in a one-pot, ecofriendly reaction. IR, NMR and XRD investigations showed that the suggested chemical structures of these materials are more stable, safe, and have higher surface areas than those of the native Ca-MMT. These synthetic nanocomposites could be promising materials in food packaging and environmentally friendly plastic applications.

## Supplementary information

Figure S1 IR of Ca-MMT.

Figure S2 IR of material 5.

Figure S3 ^1^HNMR of material 5.

Figure S4 ^13^CNMR of material 5.

Figure S5 DEPT 135 NMR of material 5.

Figure S6 IR of material 6.

Figure S7 ^1^HNMR of material 6.

Figure S8 ^13^CNMR of material 6.

Figure S9 IR of material 7.

Figure S10 ^1^HNMR of material 7.

Figure S11 ^13^CNMR of material 7.

Figure S12 DEPT 135 NMR of material 7.

Figure S13 IR of material 8.

Figure S14 ^1^HNMR of material 8.

Figure S15 ^13^CNMR of material 8.

## Figures and Tables

**Table 1:** UV–Vis spectra of materials 5–8.

Compound No	Peak		Valley	
	Wavelength nm	Abs	Wavelength nm	Abs
Compound 5	405.50,	0.117	378.00,	0.110
Compound 6	298.00, 274.00, 256.00, 247.00	0.948, 0.793 0.706 2.247	278.00, 269.00, 254.00	0.376 0.526 0.309
Compound 7	323.50	3.994	321.50,	3.675
Compound 8	298.00, 274.00, 245.00, 207.00	2.606 1.489 3.678 0.308	276.00, 268.00, 211.50	1.183 0.986 4.000

**Table 2:** D-spacing of the original (Ca-MMT) and synthesized materials as derived from XRD patterns.

Ca-MMT	Compound 5	Compound 6	Compound 7	Compound 8
15.0697Å	8.2004 Å	13.6713 Å	9.7102 Å	14.7675 Å
9.9727 Å	7.8104 Å	10.9335 Å	8.5319 Å	10.1791 Å
7.2019 Å	7.0982 Å	8.1551 Å	6.4491 Å	7.1324 Å
4.4714 Å	5.4939 Å	7.6885 Å	5.4737 Å	5.0983 Å
4.2793 Å	4.9624 Å	7.0308 Å	4.9133 Å	4.9133 Å
3.5674 Å	4.5671 Å	5.317 Å	4.4448 Å	4.458 Å
3.3459 Å	4.3796 Å	4.979 Å	4.2429 Å	4.037 Å
3.2524 Å	4.2071 Å	4.4185 Å	3.9623 Å	3.9415 Å
3.0195 Å	3.9728 Å	3.9107 Å	3.8604 Å	3.6808 Å
2.9436 Å	3.8604 Å	3.7543 Å	3.6718 Å	3.559 Å
2.8989 Å	3.6989 Å	3.5257 Å	3.453 Å	3.3312 Å
2.6947 Å	3.6015 Å	3.3166 Å	3.2665 Å	3.2179 Å
2.5687 Å	3.4849 Å	3.0315 Å	3.0135 Å	3.0255 Å
2.5185 Å	3.2665 Å	2.9898 Å	2.9211 Å	2.984 Å
2.4545 Å	3.0255 Å	2.984 Å	2.5773 Å	2.7929 Å
2.3399 Å	2.8186 Å	2.9267 Å	2.5518 Å	2.5602 Å
2.2885 Å	2.4623 Å	2.877 Å		2.5185 Å
2.2457 Å	2.3435 Å	2.7878 Å		2.4163 Å
2.1924 Å	2.2751 Å	2.6759 Å		2.372 Å
2.1652 Å	2.0396 Å	2.5433 Å		2.3364 Å
2.1301 Å	1.7203 Å	2.4941 Å		2.2818 Å
2.0266 Å		2.4276 Å		2.2425 Å
1.9986 Å		2.3364 Å		2.2203 Å
1.8991 Å		2.3329 Å		2.2172 Å
1.8179 Å		2.3122 Å		2.1534 Å
1.6921 Å		2.2393 Å		2.1244 Å
1.6419 Å		2.2203 Å		2.1046 Å
1.6164 Å		2.1742 Å		2.0369 Å
1.5411 Å		2.1563 Å		1.9812 Å
1.503 Å		2.1215 Å		1.8924 Å
1.4926 Å		2.0962 Å		1.6973 Å
1.4709 Å		1.969 Å		1.6818 Å
1.4536 Å		1.9357 Å		1.6682 Å
1.3729 Å		1.8879 Å		1.65 Å
		1.6818 Å		1.6243 Å
		1.601 Å		1.6087 Å
		1.5313 Å		1.5411 Å
		1.4978 Å		1.503 Å
		1.4634 Å		1.4887 Å
		1.4488 Å		
		1.4404 Å		
		1.4321 Å		
		1.3956 Å		
		1.3708 Å		
		1.3572 Å		
		1.4622 Å		
		1.45 Å		

**Table 3:** Principal peaks (001) position, crystallite size, and nonuniform strain of the studied materials.

Compound No	Principal peak (001)	Difference	Crystallite Size nm	Non-uniform strain %
Ca-MMT	15.0697Å		5.45	0.36
Compound 5	8.2004		10	0.08
Compound 6	13.6713Å	1.3984Å	3	0.02
Compound 7	9.7102		76	0.37
Compound 8	14.7675Å	0.3022Å	3.6	0.05

**Table 4:** BET surface area, total pore volume, average pore size, and average particle radius of the studied materials.

Organoclays	BET Surface area	Total Pore Volume	Average Pore Size	Average Particle radius
Compound 5	71.7121 m^2^/g	0.145203 cc/g	4.0496 nm	1.9015e+001 nm
Compound 6	72.7738 m^2^/g	0.141333 cc/g	3.88416 nm	1.8738e+001 nm
Compound 7	69.5468 m^2^/g	0.192609 cc/g	5.53898 nm	1.9607e+001 nm
Compound 8	63.9383 m^2^/g	0.126602 cc/g	3.96013 nm	2.1327e+001 nm

**Table 5:** Elemental composition (wt %) of Ca-MMT and materials 6 and 8 at selected regions.

	C	N	O	Si	Al	Ca	Fe	Mg	K	Ti
**Ca-MMT**	0	0	53.0	23.0	12.0	2.7	0.8	1.3	0.6	0.4
**6**	30.2	1.6	46.9	9.8	5.0	0.6	0.3	0.8	0.1	0.3
**8**	52.7	2.0	36.2	4.2	2.2	0.3	0.4	0.4	0	0

## Data Availability

Authors can confirm that all relevant data are included in the article and its supplementary information files.
