# Supplementary figures and images for: Stable mesoporous nanocomposites of iminocyclohexanones coordinated with Ca montmorillonite
Source: Turk J Chem. 2025 May 17;49(5):632–46. doi: 10.55730/1300-0527.3759 (PMC12604924; doi:10.55730/1300-0527.3759)

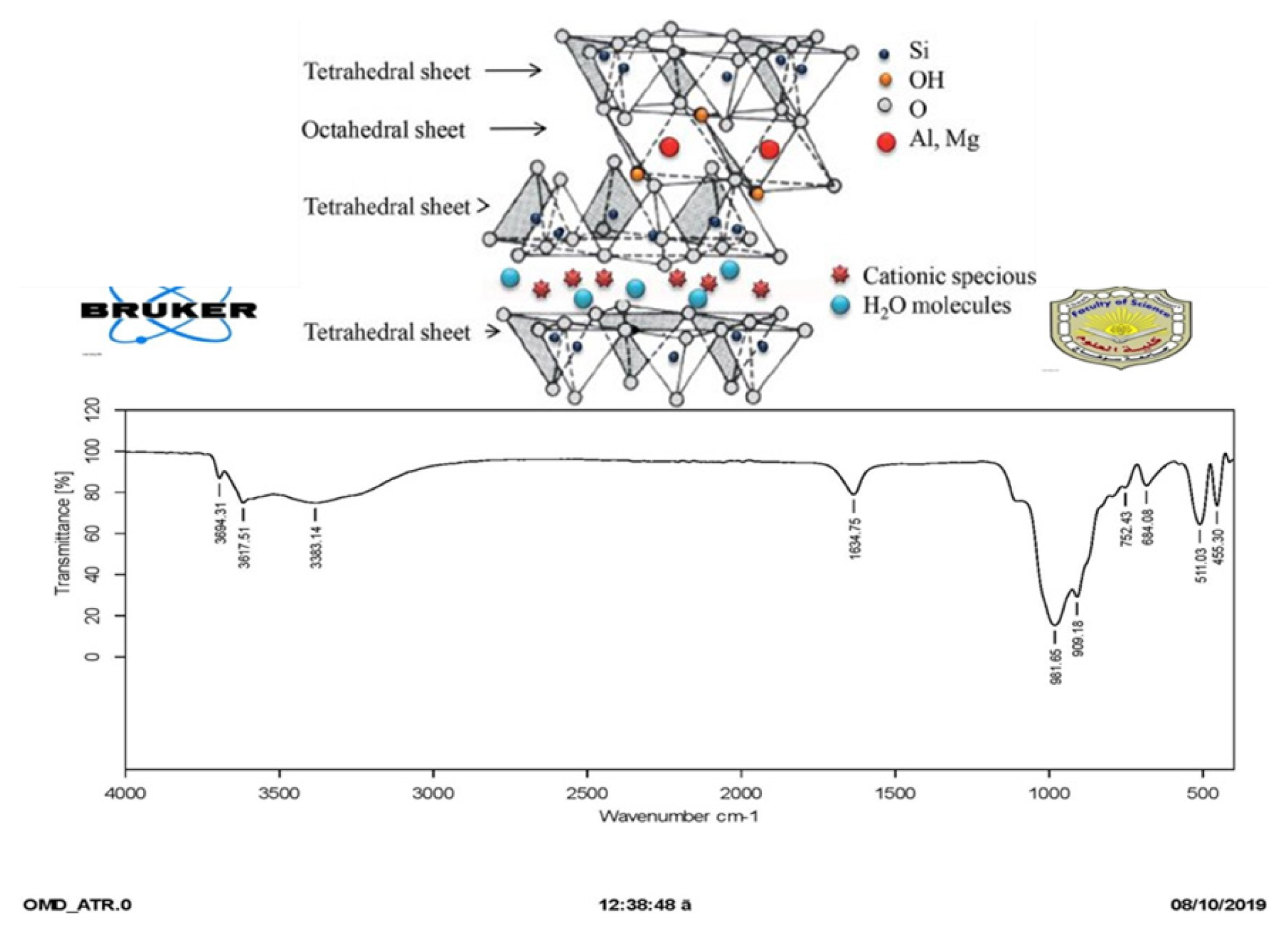

Supplement: Figure S1 — IR of Ca-MMT. [file tjc-49-05-632s1.tif]

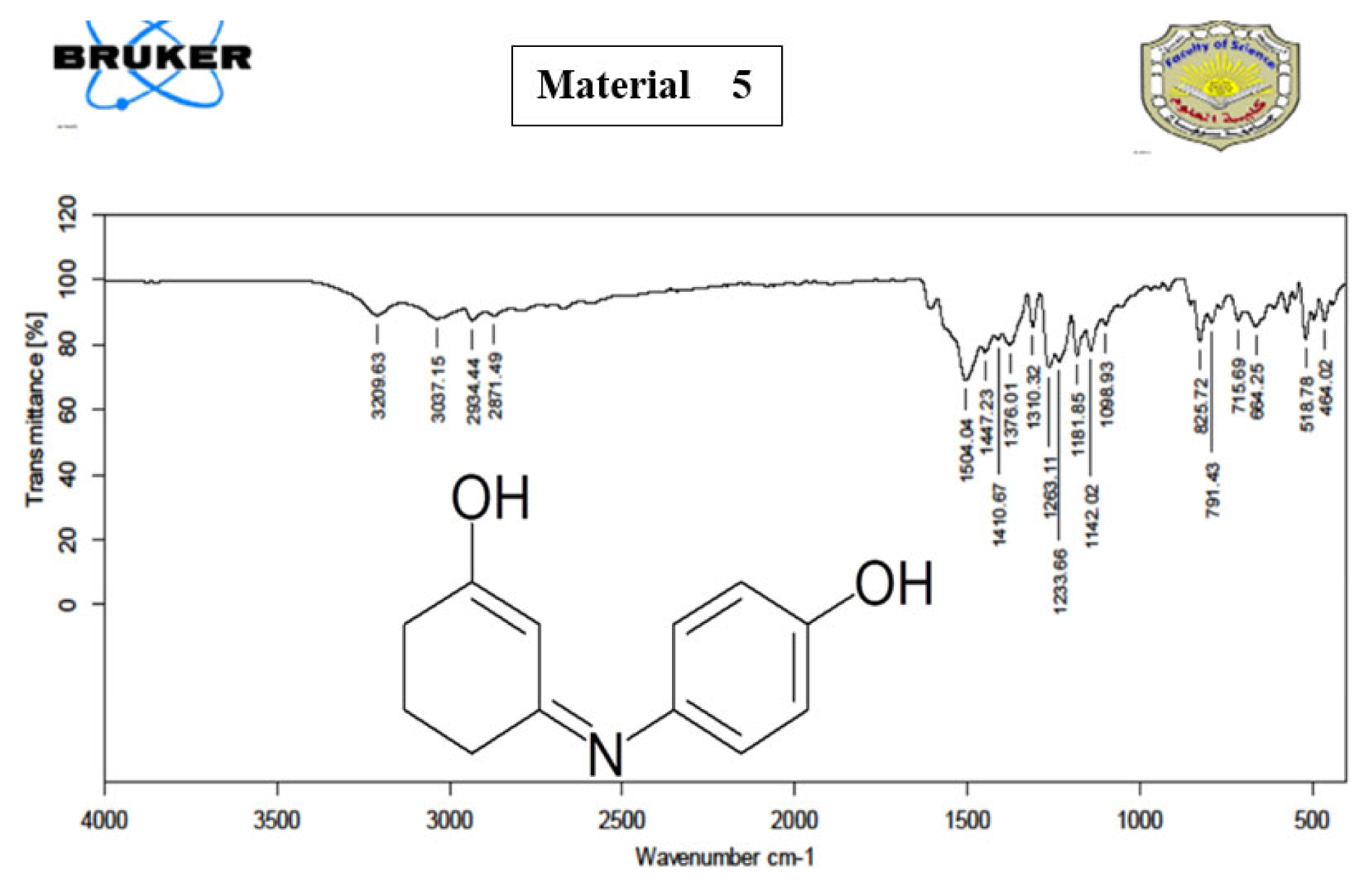

Supplement: Figure S2 — IR of material 5. [file tjc-49-05-632s2.tif]

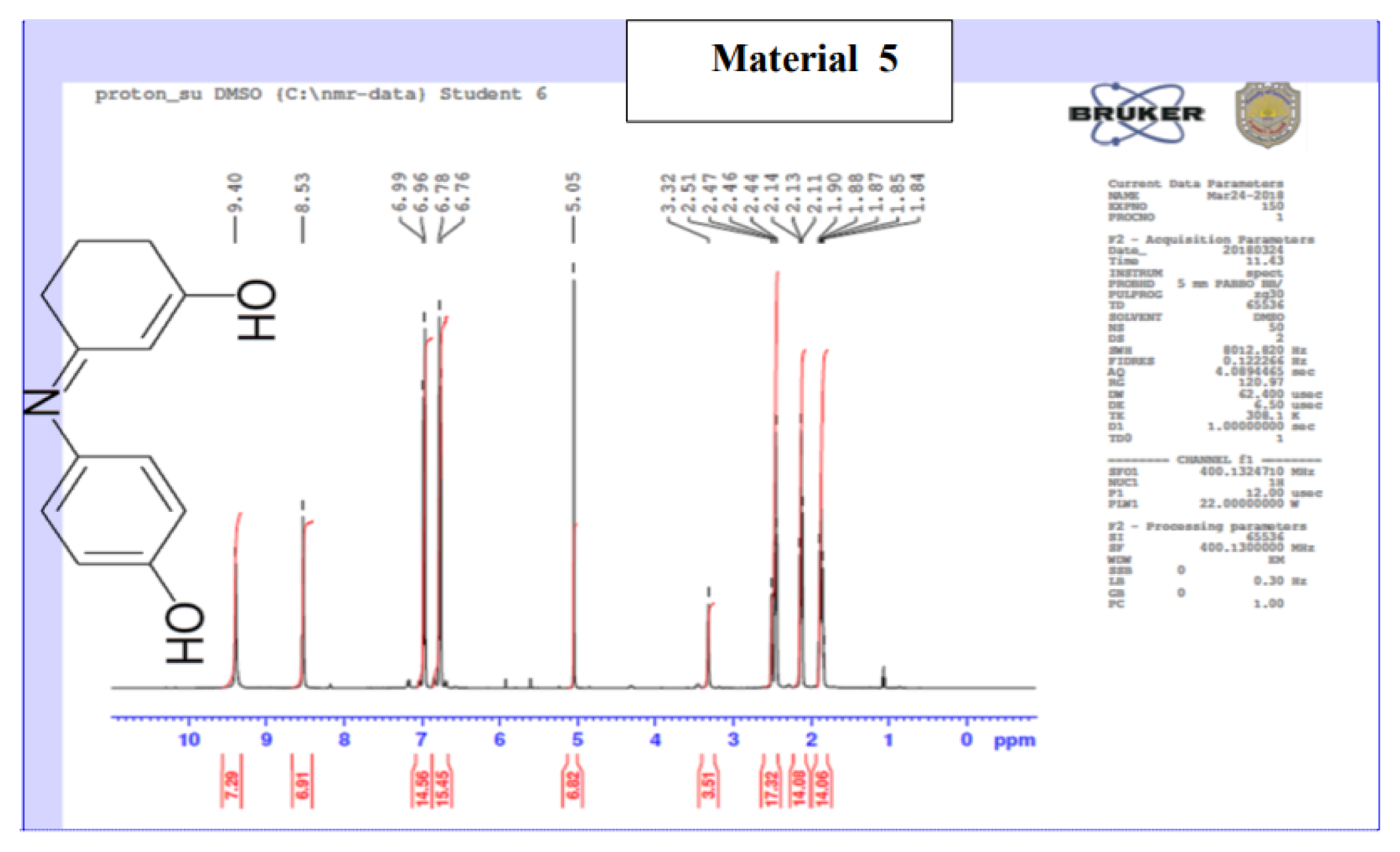

Supplement: Figure S3 — 1HNMR of material 5. [file tjc-49-05-632s3.tif]

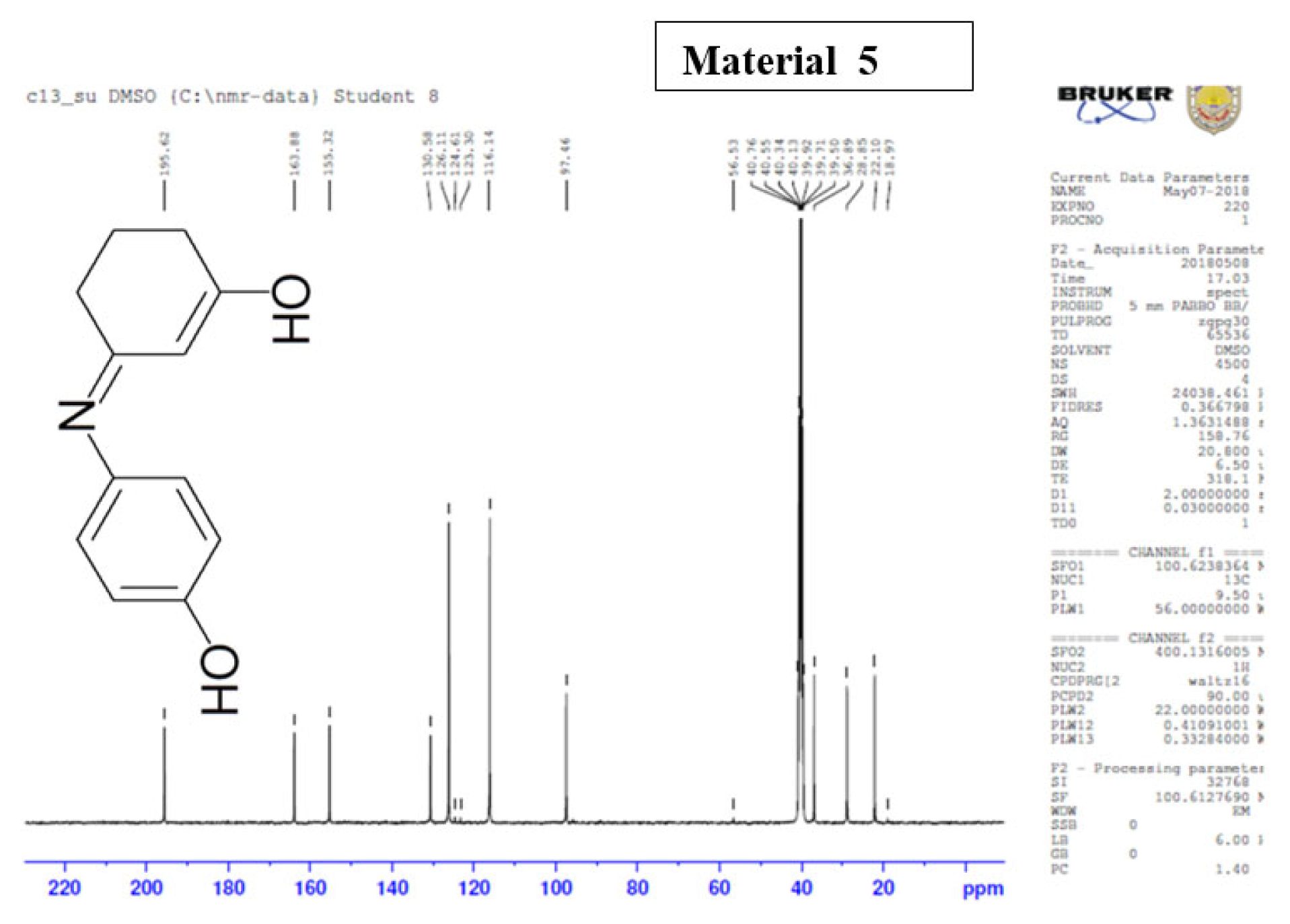

Supplement: Figure S4 — 13CNMR of material 5. [file tjc-49-05-632s4.tif]

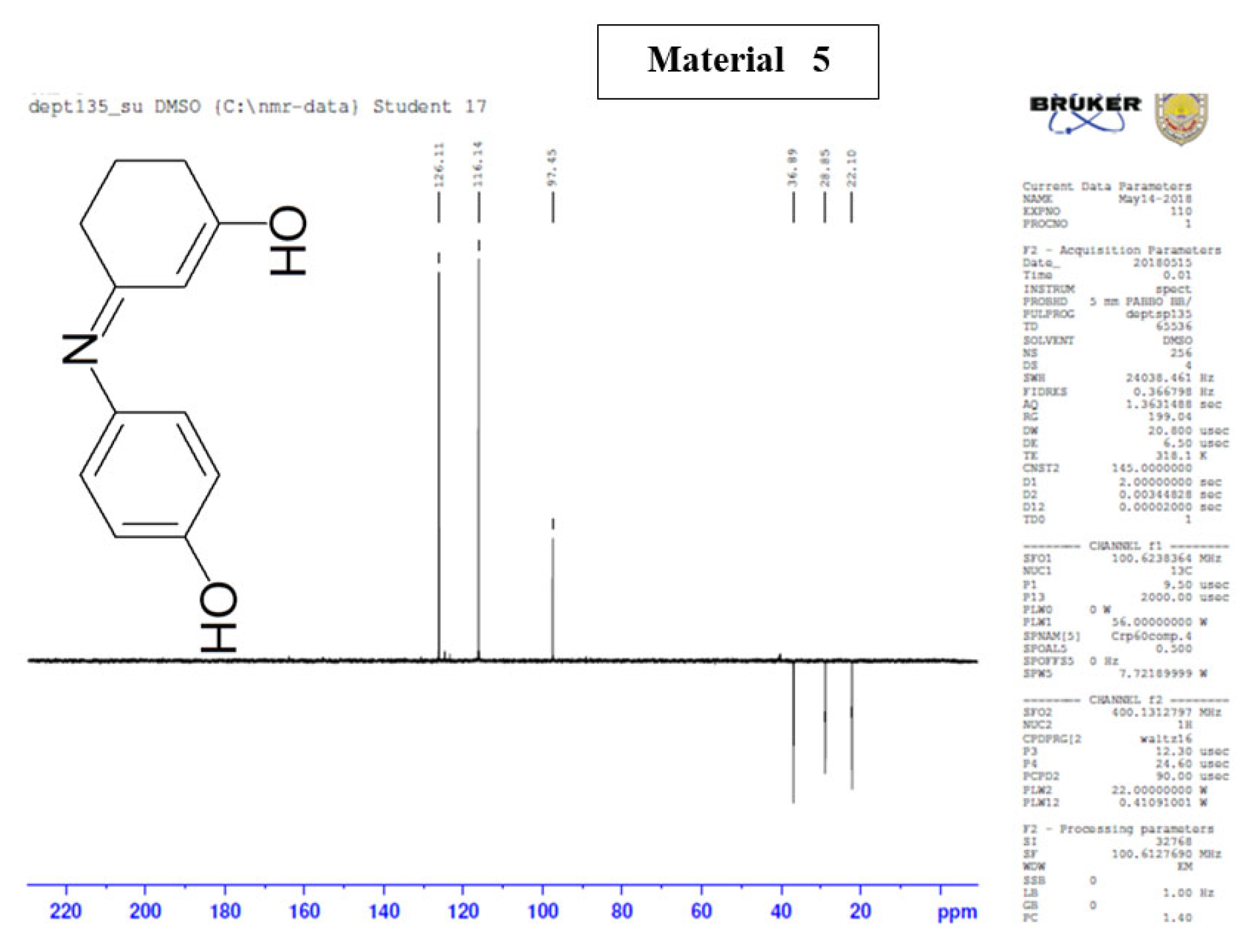

Supplement: Figure S5 — DEPT 135 NMR of material 5. [file tjc-49-05-632s5.tif]

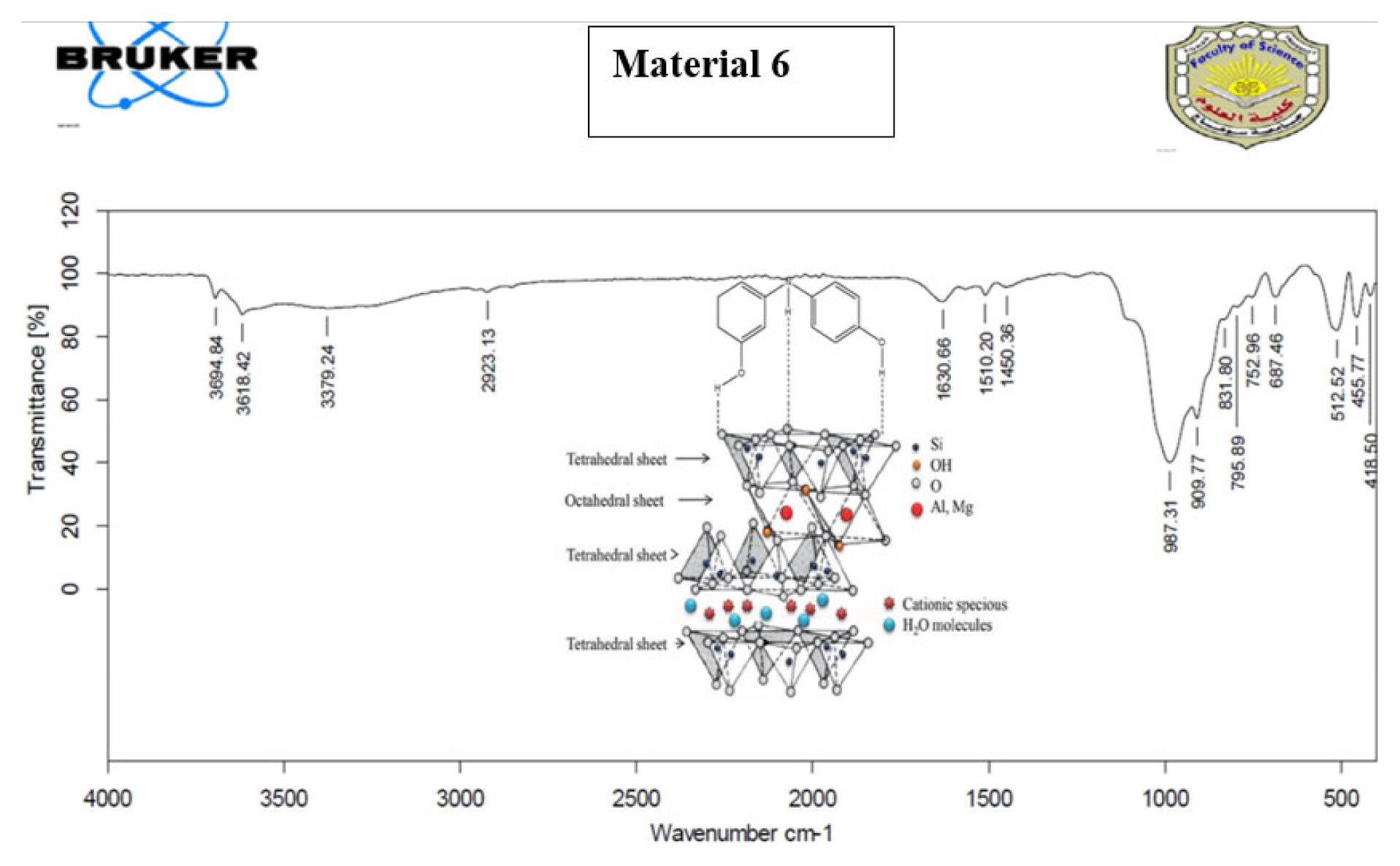

Supplement: Figure S6 — IR of material 6. [file tjc-49-05-632s6.tif]

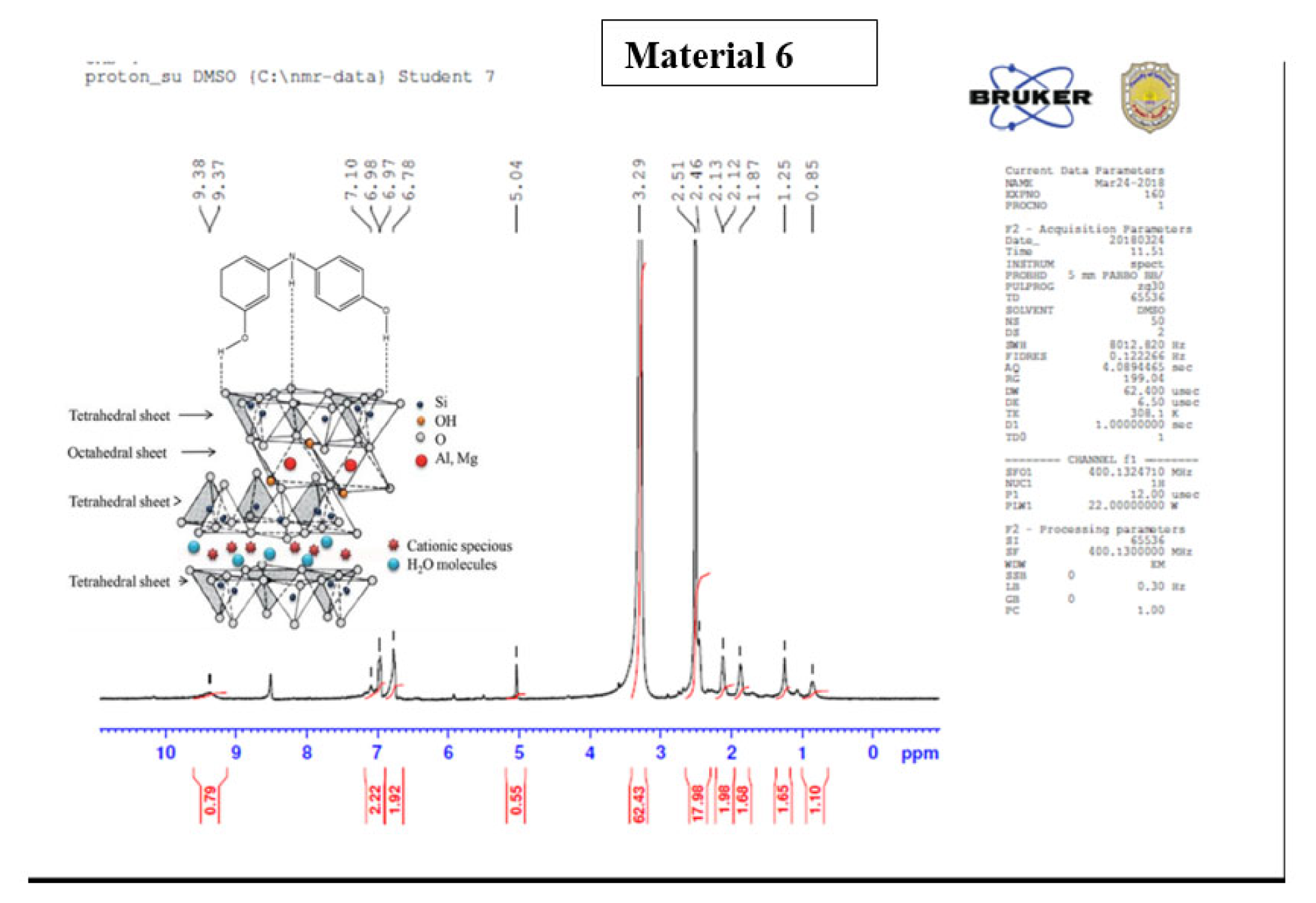

Supplement: Figure S7 — 1HNMR of material 6. [file tjc-49-05-632s7.tif]

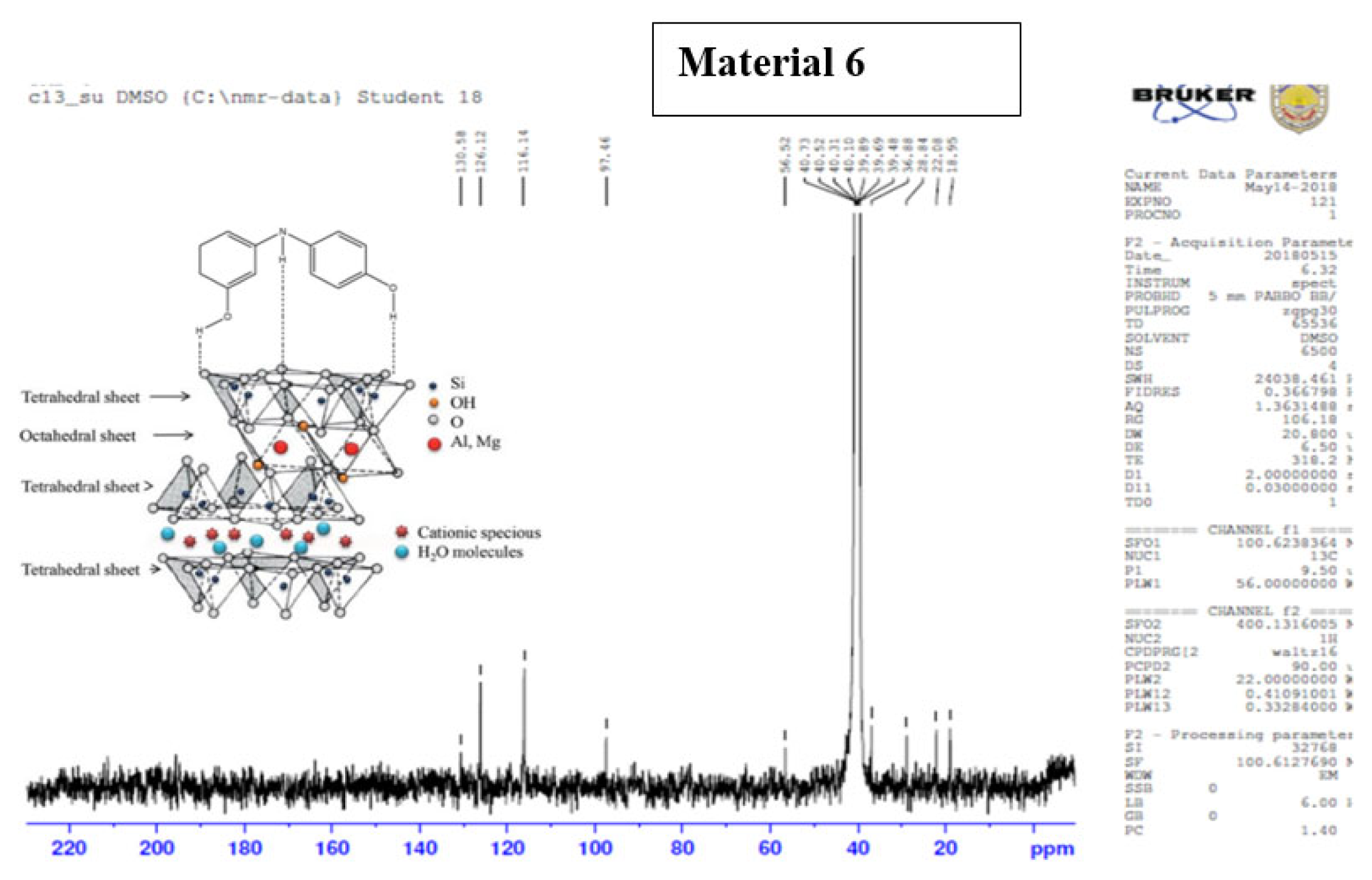

Supplement: Figure S8 — 13CNMR of material 6. [file tjc-49-05-632s8.tif]

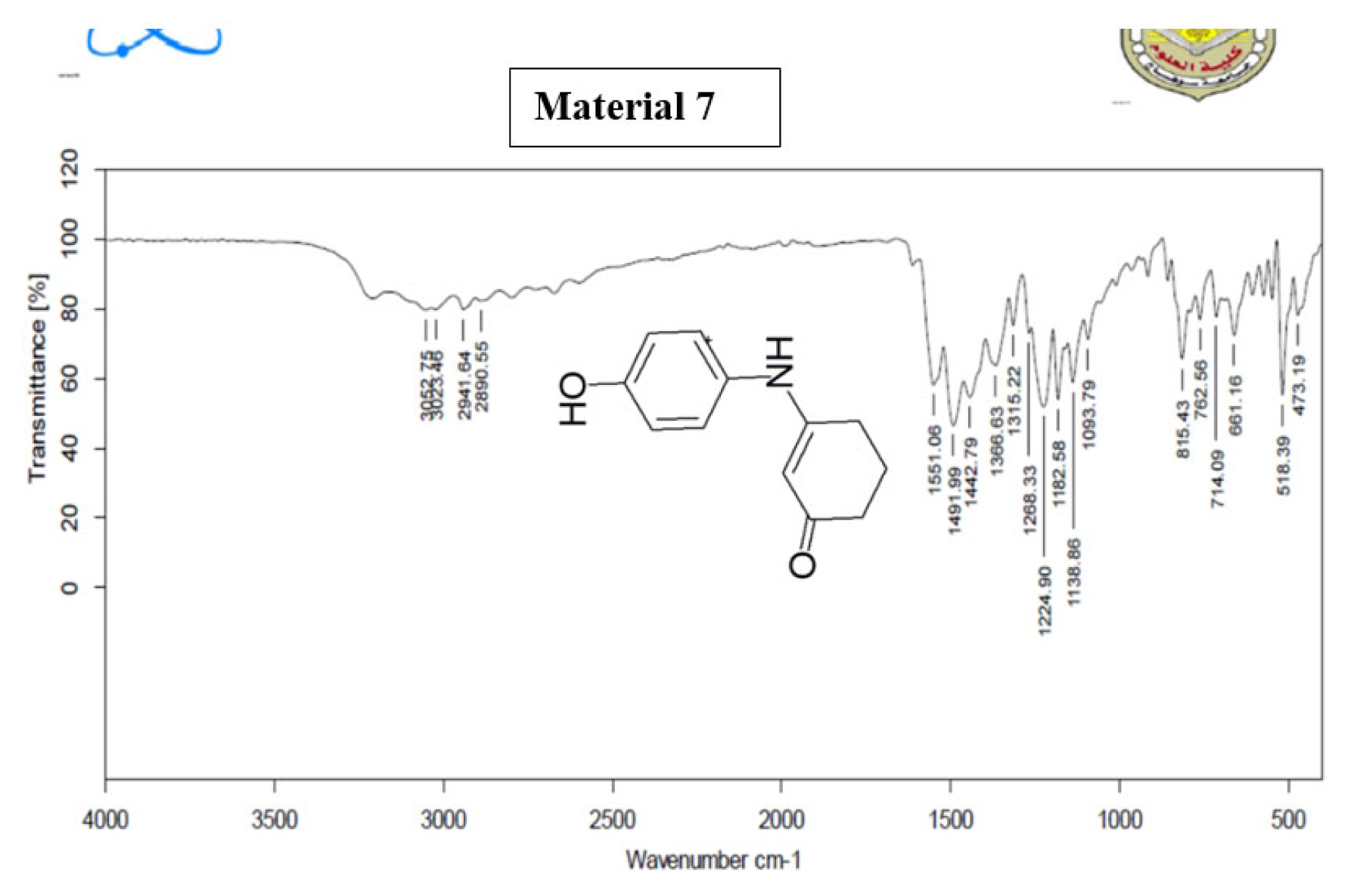

Supplement: Figure S9 — IR of material 7. [file tjc-49-05-632s9.tif]

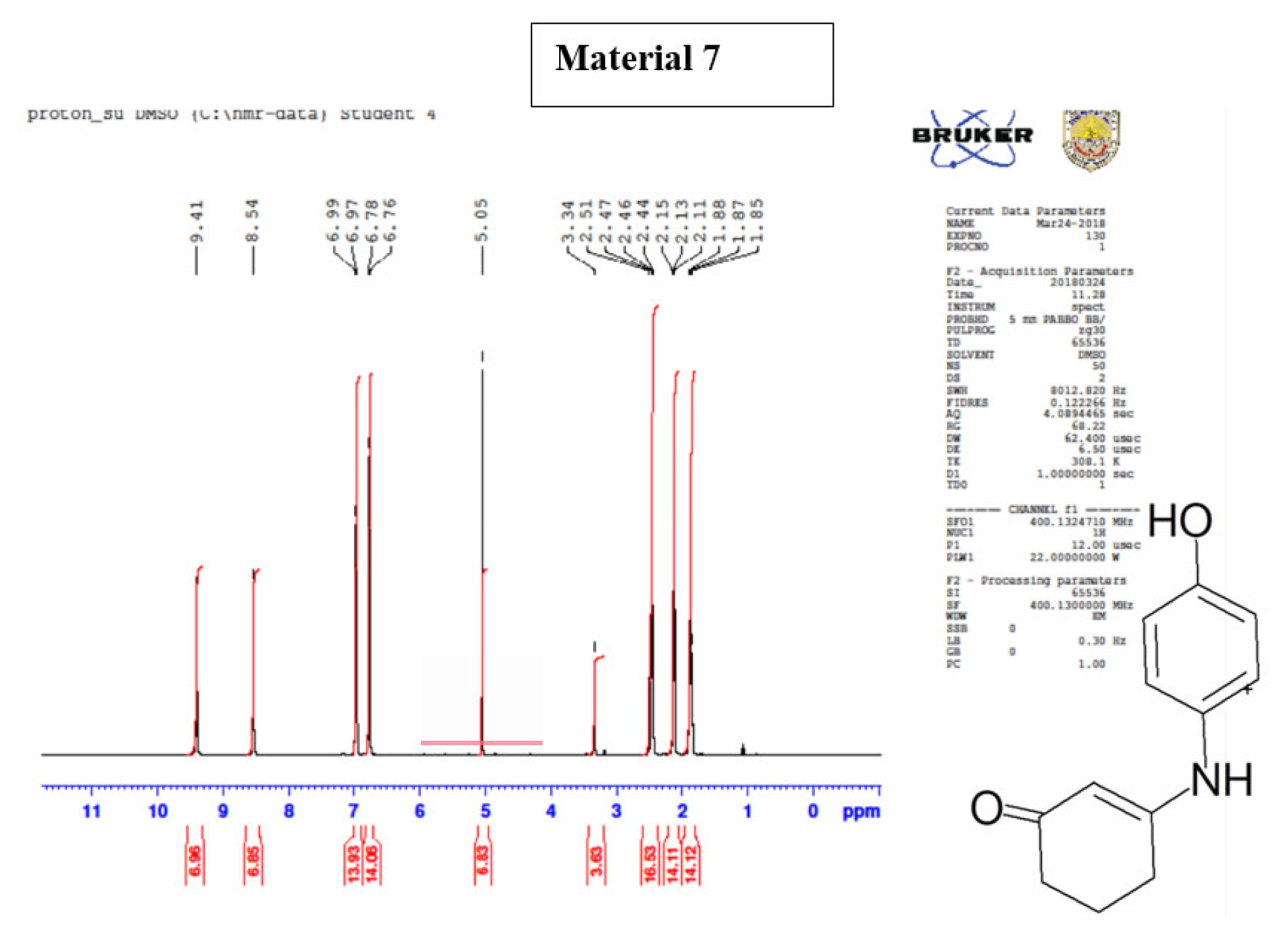

Supplement: Figure S10 — 1HNMR of material 7. [file tjc-49-05-632s10.tif]

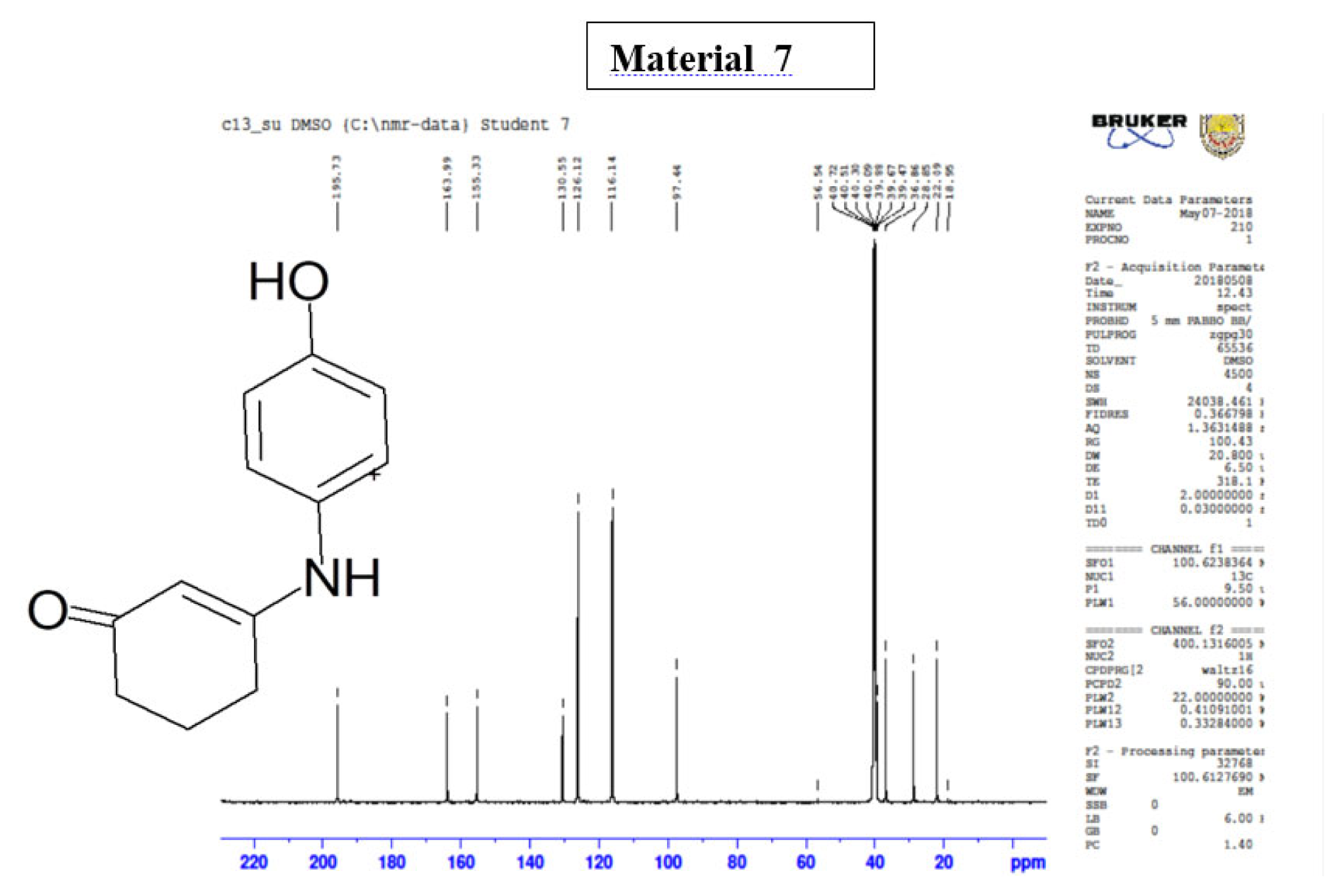

Supplement: Figure S11 — 13CNMR of material 7. [file tjc-49-05-632s11.tif]

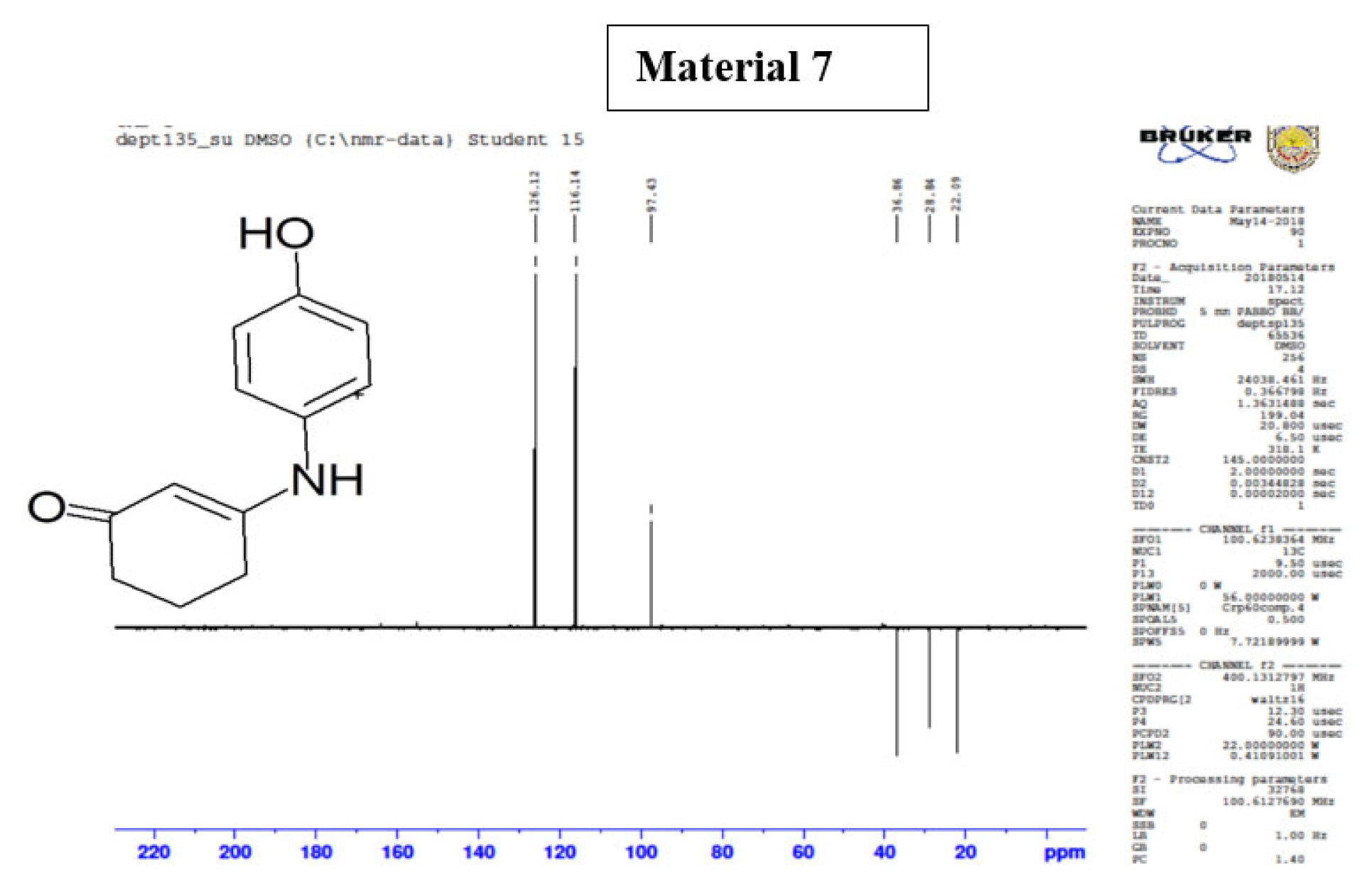

Supplement: Figure S12 — DEPT 135 NMR of material 7. [file tjc-49-05-632s12.tif]

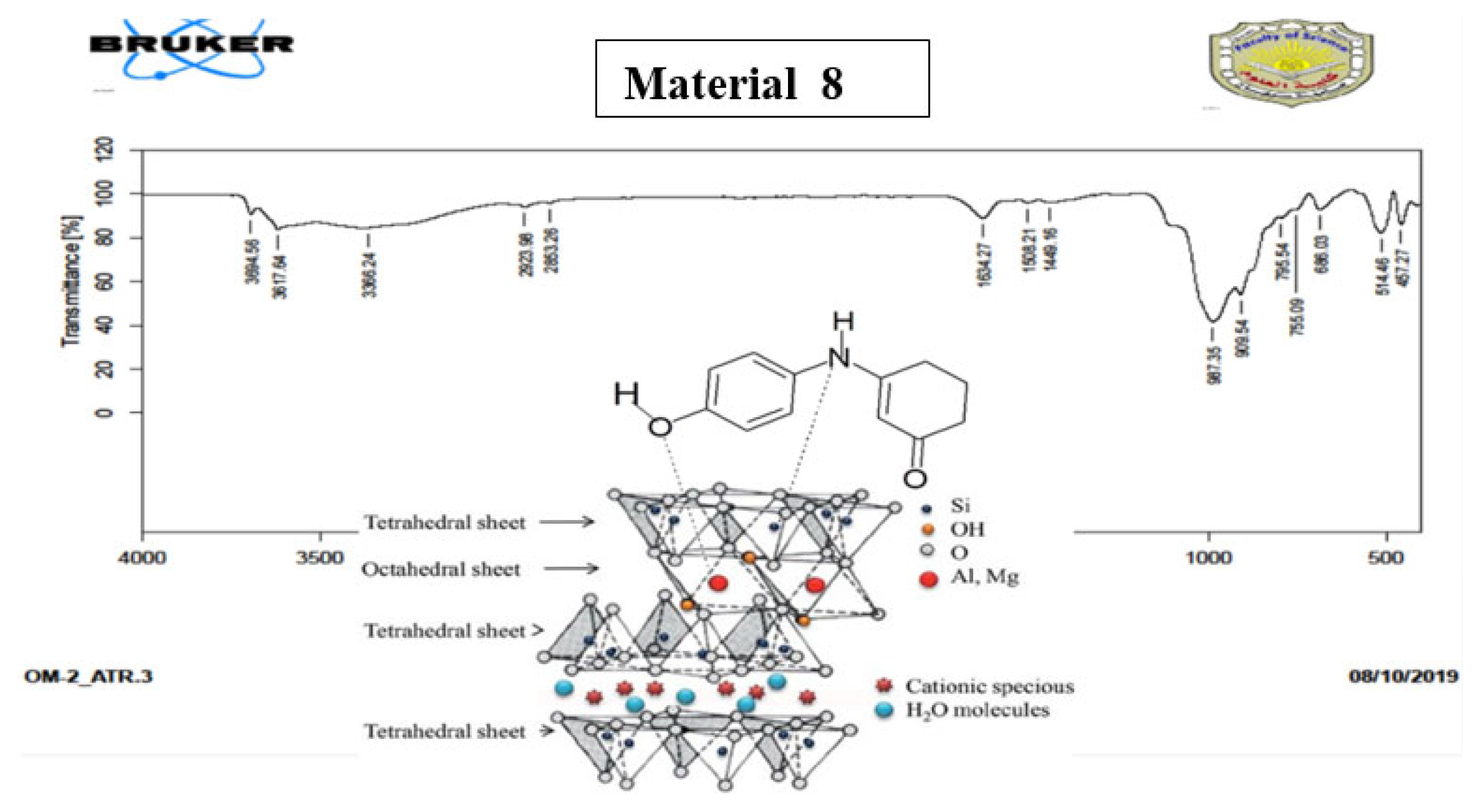

Supplement: Figure S13 — IR of material 8. [file tjc-49-05-632s13.tif]

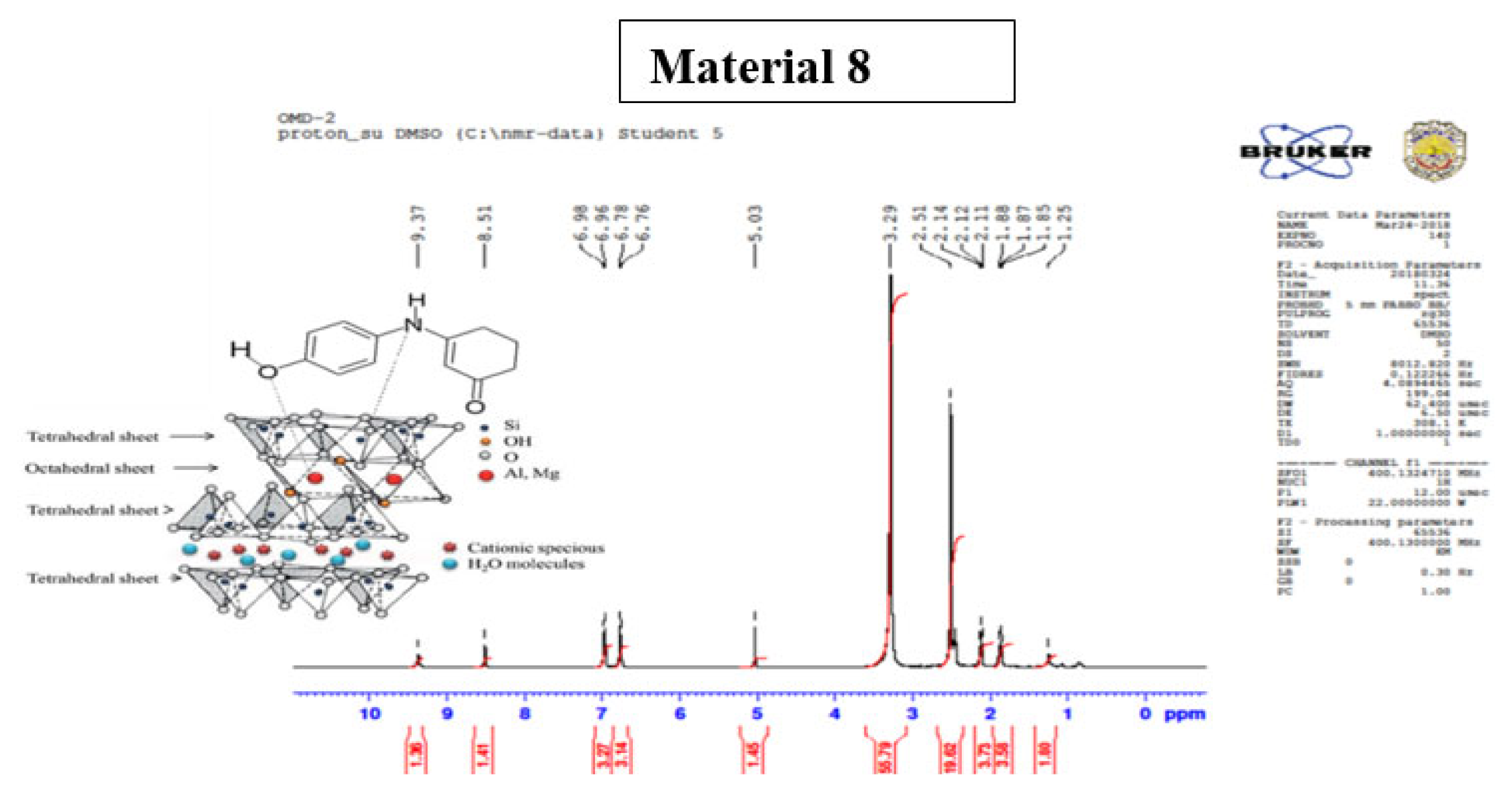

Supplement: Figure S14 — 1HNMR of material 8. [file tjc-49-05-632s14.tif]

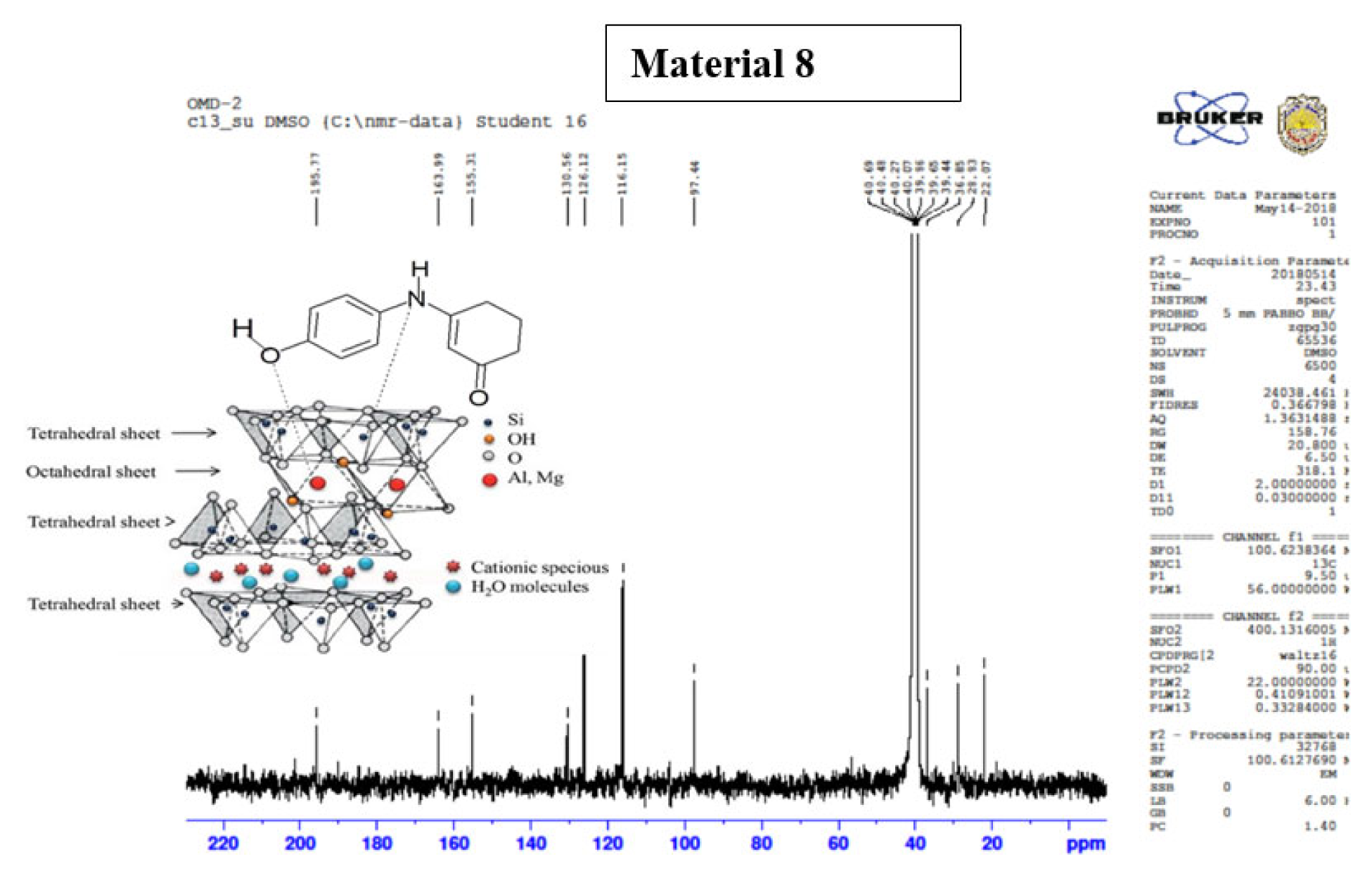

Supplement: Figure S15 — 13CNMR of material 8. [file tjc-49-05-632s15.tif]
